# Integrated spatial multiomics reveals fibroblast fate during tissue repair

**DOI:** 10.1073/pnas.2110025118

**Published:** 2021-10-07

**Authors:** Deshka S. Foster, Michael Januszyk, Kathryn E. Yost, Malini S. Chinta, Gunsagar S. Gulati, Alan T. Nguyen, Austin R. Burcham, Ankit Salhotra, R. Chase Ransom, Dominic Henn, Kellen Chen, Shamik Mascharak, Karen Tolentino, Ashley L. Titan, R. Ellen Jones, Oscar da Silva, W. Tripp Leavitt, Clement D. Marshall, Heather E. des Jardins-Park, Michael S. Hu, Derrick C. Wan, Gerlinde Wernig, Dhananjay Wagh, John Coller, Jeffrey A. Norton, Geoffrey C. Gurtner, Aaron M. Newman, Howard Y. Chang, Michael T. Longaker

**Affiliations:** ^a^Hagey Laboratory for Pediatric Regenerative Medicine, Division of Plastic and Reconstructive Surgery, Stanford University School of Medicine, Stanford, CA 94305;; ^b^Department of Surgery, Stanford University School of Medicine, Stanford CA 94305;; ^c^Center for Personal Dynamic Regulomes, Stanford University, Stanford, CA 94305;; ^d^Institute for Stem Cell Biology and Regenerative Medicine, Stanford University School of Medicine, Stanford, CA 94305;; ^e^Stanford Functional Genomics Facility, Stanford University, Stanford, CA 94305;; ^f^Department of Biomedical Data Science, Stanford University, Stanford, CA 94305;; ^g^HHMI, Stanford University, Stanford, CA 94305

**Keywords:** spatial epigenomics, spatial transcriptomics, multiomics, fibrosis, chromatin accessibility

## Abstract

In the skin, tissue injury results in fibrosis in the form of scars composed of dense extracellular matrix deposited by fibroblasts. The therapeutic goal of regenerative wound healing has remained elusive, in part because principles of fibroblast programming and adaptive response to injury remain incompletely understood. Here, we present a multimodal -omics platform for the comprehensive study of cell populations in complex tissue, which has allowed us to characterize the cells involved in wound healing across both time and space. We employ a stented wound model that recapitulates human tissue repair kinetics and multiple Rainbow transgenic lines to precisely track fibroblast fate during the physiologic response to skin injury. Through integrated analysis of single cell chromatin landscapes and gene expression states, coupled with spatial transcriptomic profiling, we are able to impute fibroblast epigenomes with temporospatial resolution. This has allowed us to reveal potential mechanisms controlling fibroblast fate during migration, proliferation, and differentiation following skin injury, and thereby reexamine the canonical phases of wound healing. These findings have broad implications for the study of tissue repair in complex organ systems.

## Significance

In the skin, tissue injury results in fibrosis in the form of a scar composed of dense extracellular matrix deposited by fibroblasts. Therapies that promote tissue regeneration rather than fibrosis remain elusive because principles of fibroblast programming and response to injury remain incompletely understood. Here, we present a multimodal -omics platform for the study of cell populations in complex tissue, which has allowed us to characterize wound healing fibroblasts across both time and space. We identify functionally distinct fibroblast subpopulations and track cell fate during the response to wounding. We demonstrate that populations of fibroblasts migrate, proliferate, and differentiate in an adaptive response to disruption of their environment. These results illustrate fundamental principles underlying the cellular response to tissue injury.

Tissue fibrosis and its sequelae are associated with 45% of all mortality in the United States (1, 2). In the skin, wound healing is achieved through fibrosis and formation of a scar, which is composed of dense extracellular matrix. Scars are stiff, have poor vascularization, lack normal skin appendages, and accordingly are devoid of the skin’s native functionality. As a result, scars can result in lifelong disability secondary to disfigurement and dysfunction (3). Fibroblasts are the cells responsible for deposition of scar tissue. While several studies have characterized subtypes of fibroblasts involved in wound healing, the development of novel therapies that foster regeneration (rather than fibrosis) has remained limited because the origins, heterogeneity, and behavior of fibroblasts during tissue repair are not yet comprehensively understood.

Current knowledge of wound biology is largely derived from experiments performed in mice. However, translating cutaneous tissue repair in mice to humans is challenging due to species-specific anatomical differences. The panniculus carnosus is a subdermal muscle layer found throughout the body of mice that substantially contracts in response to wounding, enabling wound closure primarily through contracture of the mouse’s loose skin. In humans, an analog to this muscle exists only in the neck (the platysma muscle), the hand (palmaris brevis), and the scrotum (dartos muscle). Fibroblast heterogeneity has been previously explored in wound healing using mouse models in which large, unstented wounds (1.5-cm diameter) heal primarily by contraction, with only a small portion in the center healing through reepithelialization and deposition of connective tissue from fibroblasts (the primary mechanism of wound healing in humans) (4, 5). To recapitulate clinically relevant wound healing using mouse models, we utilize a stented wound model, which limits contraction of the panniculus carnosus and thereby mimics the wound healing kinetics of tight-skinned humans (6). Given that local tissue mechanics play a central role in scar formation (7–9), this model permits us to interrogate fibroblast mechanobiology in a more clinically relevant manner.

Recent advances in sequencing and cell capture technology have enabled the assessment of gene expression with reference to tissue organization using spatial transcriptomics. This approach has only been applied to a limited number of tissue types to date, primarily in the study of tumors, including prostate cancer (10), skin cancer (11, 12), and breast cancer (13), as well as bone marrow (14), joints (15), and brain tissue (16). However, to our knowledge, spatial transcriptomic analysis over time has yet to be applied to characterize wound healing. Moreover, the spatial and temporal distributions of the single-cell chromatin landscapes underlying gene expression have yet to be described.

Here, using transgenic mouse models, we assess the proliferation of local, tissue-resident fibroblast cells in wound healing. By establishing a microsurgical approach to independently isolate fibroblasts from spatially distinct regions within the wound, we interrogate Rainbow-labeled fibroblasts from critical timepoints during the course of wound closure. The Rainbow mouse model is a four-color reporter system that permits precise clonal analysis and lineage tracing. Using this model with phenotype-paired single-cell RNA and ATAC sequencing (scRNA-seq and scATAC-seq), we are able to define the spatial and temporal heterogeneity of wound fibroblasts with unique granularity. Using full-length, plate-based scRNA-seq, we assess the differentiation states of individual cells as they proliferate and migrate from the outer wound region inward (17). By disrupting this process using small molecule inhibition or genetic knockdown of focal adhesion kinase (FAK, *Ptk2*), we further elucidate the relationship between wound healing fibroblast activation and microenvironmental cues. By integrating our scRNA-seq and scATAC-seq analyses using the recently developed ArchR platform (18), we delineate interrelated changes in chromatin accessibility and gene expression driving wound closure and fibrosis and identify distinct wound fibroblast subpopulations. Furthermore, using CIBERSORTx deconvolution (19) of bulk RNA-seq data, we are able to categorize a putative fibroblast subpopulation-based response to local tissue injury. Finally, we introduce spatial multiomics, combining spatial transcriptomics with paired scRNA-seq and scATAC-seq datasets to impute spatial epigenomic properties and map chromatin accessibility states in the healing wound. Collectively, this work defines the spatial and temporal dynamics of the fibroblast response to injury and provides a multimodal -omics framework for future studies in tissue repair.

## Results

### Wounding Triggers Polyclonal Proliferation of Tissue-Resident Fibroblasts.

**Fig. 1.**
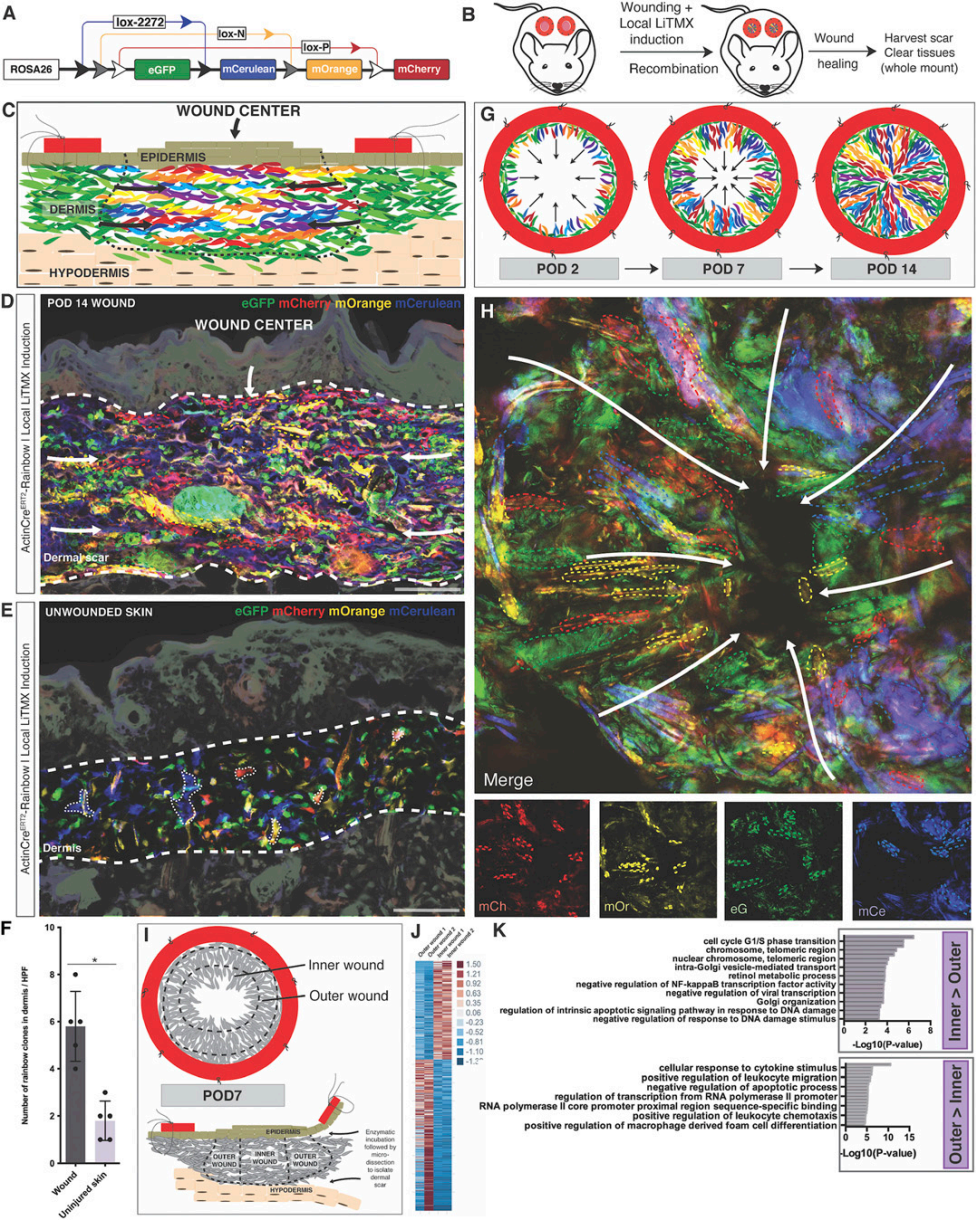
Wounding triggers polyclonal proliferation of tissue-resident fibroblasts. (*A*) Schematic of the Rainbow mouse construct. (*B*) Schematic showing wound healing model using Rainbow mice with local Cre recombinase induction using 4-hydroxytamoxifen liposomes (LiTMX). (*C*) Schematic showing a Rainbow wound cross-section. Black dotted line highlights wound scar area; arrows indicate the direction of cellular proliferation during wound healing. Structures are as labeled. (*D*) Representative confocal image of POD 14 wound cross-sections from *Actin-Cre*^*ERT2*^*::Rosa26*^*VT2/GK3*^ mice induced locally with LiTMX at the time of wound creation. Thick white dotted lines highlight scar boundaries. Individual Rainbow cell clones are highlighted with thin colored dotted lines. Arrows indicate direction of wound healing. *n > 5.* (Scale bar, 50 μm.) (*E*) Representative confocal images of unwounded skin from *Actin-Cre*^*ERT2*^*::Rosa26*^*VT2/GK3*^ mice induced locally with LiTMX. Thick white dotted lines highlight dermal boundaries. Individual Rainbow cell clones are highlighted with thin white dotted lines. *n > 5.* (Scale bar, 50 μm.) (*F*) Rainbow clone counts in wounds versus uninjured skin. *n* = 5 per condition. *P < 0.05. (*G*) Schematic of dorsal, stented, excisional wound healing in the Rainbow mouse model (whole-mount view), with polyclonal proliferation of Rainbow fibroblasts from the outer wound edge inward across time from POD 2 (*Left*), to POD 7 (*Middle*), to POD 14 (*Right*). Black arrows highlight the apparent direction of proliferation. (*H*) Representative confocal imaging of a POD 14 whole-mounted wound harvested from *Actin-Cre*^*ERT2*^*::Rosa26*^*VT2/GK3*^ mice showing the polyclonal proliferation of wound fibroblasts radially toward the center of the wound (dark area at center). White arrows highlight the direction of cell proliferation; individual cell clones are highlighted with thin colored dotted lines. *Bottom* subpanels denote individual Rainbow color contributions to merged image. mCh, membrane (m)Cherry; mOr, mOrange; mCe, mCerulean; eG, eGFP. *n* > 5. (*I*) Schematics illustrating microdissection strategy for isolation of inner and outer wound regions (*Top*), followed by enzymatic separation of the dermal scar from the epi- and hypodermis (*Bottom*). (*J*) Heatmap displaying expression data for genes significantly different between POD 7 inner and outer region wound fibroblasts. Legend at *Right* displays fold change. (*K*) Gene Ontology (GO) enrichment analysis comparing gene expression data from POD 7 inner and outer region wound fibroblasts. *Top* shows GO biological processes up-regulated in inner region fibroblasts compared with outer region fibroblasts, while the *Bottom* shows the same for outer region fibroblasts compared with inner. Top 10 most significant gene sets are displayed for each condition.

To explore the lineage dynamics of wound fibroblasts, we examined stented wound healing using the Rainbow (*Rosa26*^*VT2/GK3*^) mouse model (20). Rainbow mice contain a transgenic four-color reporter construct in the *Rosa26* locus. Upon induction with Cre recombinase, the four colors irreversibly recombine such that all progeny cells will have the same color as their parent cells, thereby permitting stochastic lineage tracing and clonal analysis (Fig. 1*A*). We developed a technique for local induction using activated tamoxifen liposomes (LiTMX) in order to induce reporter recombination exclusively in tissue-resident cells (Fig. 1*B*) (21). Following injury, local skin fibroblasts were found to proliferate in a linear, polyclonal manner along the cross-sectional wound interface (Fig. 1 *C* and *D*), whereas fibroblasts in uninjured skin exhibited minimal clonality (Fig. 1 *E* and *F*). These data support the presence of local cells that are activated in response to injury and proliferate polyclonally to fill the wound “gap.”

Many cell surface and lineage markers have been associated with fibroblasts involved in wound healing, including *Pdgfra*, Engrailed-1 (*En1*), and CD26 (*Dpp4*) (22–24). However, we and others have found expression of such markers to be variable throughout wound tissue (*SI Appendix*, Fig. S1*A*), suggesting spatial and functional heterogeneity among the fibroblasts that respond to injury. We asked whether there might be one or more fibroblasts activated following injury that could give rise to more diverse downstream fibroblast phenotypes. If so, we wondered whether such cells would be of tissue-resident origin, as suggested by previous studies (25–27) (*SI Appendix*, Fig. S1*B*), or originate from peripheral circulation. To explore this, we employed transgenic parabiotic mice in conjunction with the splinted excisional wound healing model described above (6) (*SI Appendix*, Fig. S1*C*). eGFP donor mice were parabiosed to wild-type (*C57BL/6J*) mice (*SI Appendix*, Fig. S1*D*). A shared blood supply was established by 2 wk after surgery (*SI Appendix*, Fig. S1*E*), at which time wounds were made on the dorsum of each wild-type parabiont. Wounds were then harvested at postoperative day (POD) 7 (midway through healing) or POD 14 (when the wound has fully reepithelialized). While systemically infiltrating GFP^+^ cells were found in wild-type mouse wounds at both timepoints, the overwhelming majority (>80%) of GFP^+^ cells were also CD45^+^ and thus of hematopoietic (nonfibroblast) lineage (*SI Appendix*, Fig. S1*F*). These data further support the growing body of literature indicating that the fibroblasts responsible for wound healing are local, tissue-resident cells (25–27).

Returning to the Rainbow mouse model, we developed a tissue clearing and whole-mount protocol to visualize wound healing biology with the Rainbow mouse (28). Using these methods in conjunction with a ubiquitous *Actin-Cre*^*ERT2*^ driver, we observed that cells were activated along the wound edge and proliferated inward in a distinct radial pattern (Fig. 1 *G* and *H*).

### Bulk Transcriptomic Analysis of Injury-Responsive Fibroblasts.

Based on the pattern of clonal proliferation extending from the outer wound edge inward, we developed a microsurgical technique to separately isolate the “inner” and “outer” components of the wound dermis (Fig. 1*I*). We isolated wound fibroblasts from these two regions at POD 7 (midpoint of healing) and unwounded skin for bulk RNA-seq evaluation. Clear differences in the gene expression profiles of inner versus outer wound fibroblasts were identified (Fig. 1 *J* and *K* and *SI Appendix*, Fig. S2 *A*–*C*), including differences in mechanotransduction and cell cycle pathways. Furthermore, we observed that inner wound fibroblasts were transcriptionally more divergent from uninjured skin than were outer wound fibroblasts (*SI Appendix*, Fig. S2*A*). These findings support broad regional differences in the proliferation and activation status of fibroblasts in the healing wound; however, these methods are limited by the lack of granularity inherent in bulk transcriptional analysis.

### Traditional Cell Surface Markers Are Not Sufficient to Characterize Regional Heterogeneity among Wound Healing Fibroblasts.

**Fig. 2.**
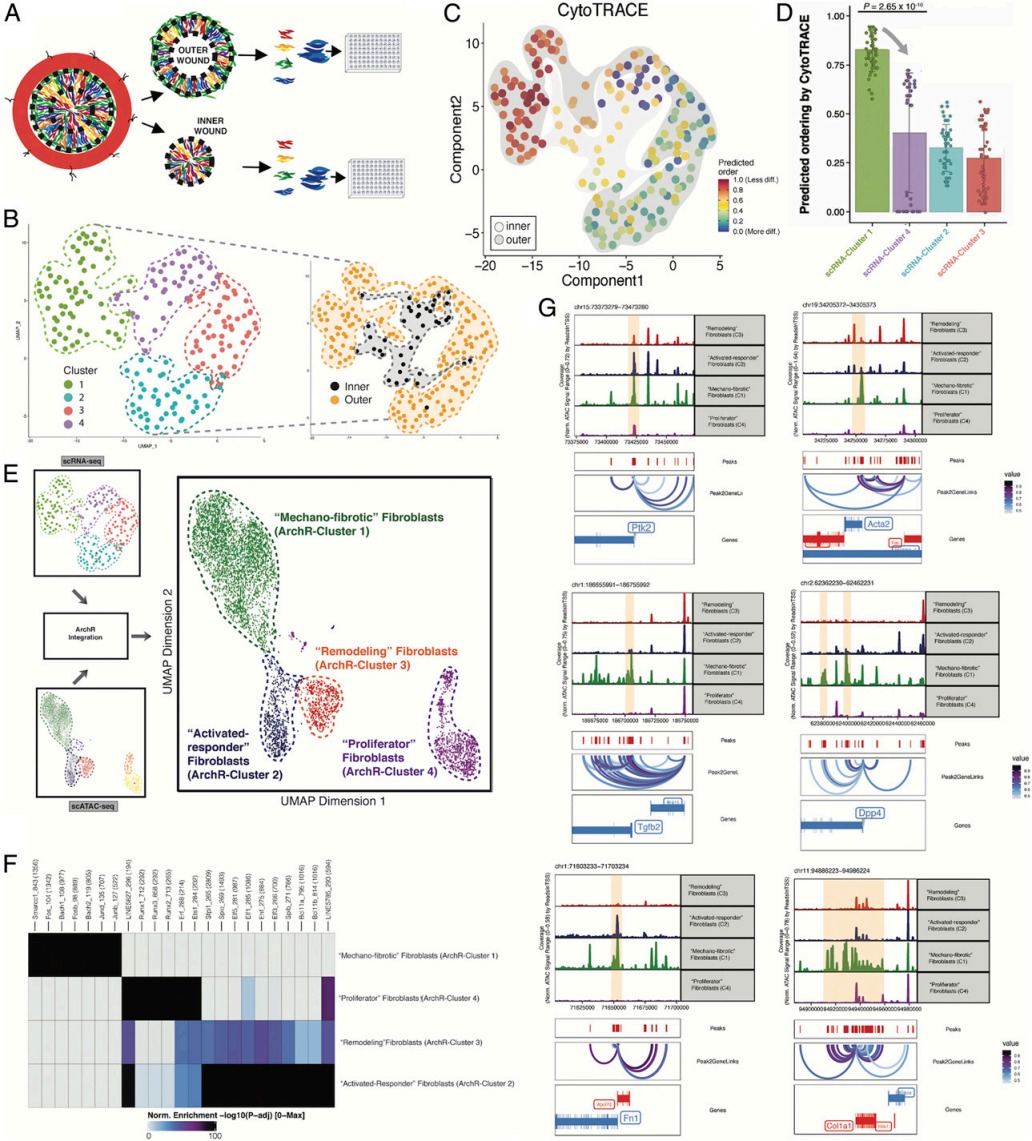
Single-cell transcriptomic and chromatin accessibility analyses delineate mechanoresponsive fibroblast subpopulations. (*A*) Schematic illustrating single-cell (sc) isolation of Rainbow wound fibroblasts from inner and outer wound regions (highlighted with black dotted lines). For scRNA-seq, mCerulean^+^ fibroblasts were arbitrarily selected from the available Rainbow colors and used for the remaining experiments in this figure. (*B*) (*Left*) Uniform manifold approximation and projection (UMAP) embedding showing scRNA-seq data from mouse wound fibroblasts FACS isolated using a lineage-negative sort strategy (29) from POD 2, POD 7, and POD 14, digitally pooled and clustered in a manner agnostic to POD and inner versus outer wound regions. Four unique fibroblast clusters were identified (clusters 1 through 4). Dotted lines highlight individual cluster distributions. (*Right*) Recoloring of *Left* UMAP plot based on fibroblast tissue region: inner (black) versus outer (orange). (*C*) CytoTRACE analysis of scRNA-seq data using the UMAP embedding from *F*. Shading indicates inner (light gray) versus outer (dark gray) wound regions. (*D*) Box plots showing the predicted ordering by CytoTRACE for individual cells within the four scRNA-seq clusters. Gray arrow indicates direction of predicted differentiation from scRNA-seq cluster 1 to cluster 4 (which corresponds to outer-to-inner wound region expansion). *P* value was derived from two-sided Student’s *t* test. (*E*) scATAC-seq evaluation of Rainbow mouse wound fibroblasts isolated in parallel with our scRNA-seq experiments (*SI Appendix*, *Methods*), integrated using the ArchR toolkit with default Louvain parameters (18) to delineate four unique multimodal fibroblast clusters. (*F*) Heatmap of scATAC-seq motifs highlighting key gene loci differentially open or closed in putative fibroblast subpopulations. (*G*) Genome tracking plots showing scATAC-seq peaks for pseudobulk replicates generated for each cluster. Associations between the peaks with fibrosis and mechanotransduction-related genes (Peak2GeneLinks) are included at the *Bottom* of each plot. Pale orange shading highlights differentially expressed peaks across the scATAC clusters. All highlighted peaks demonstrated statistically significant differential expression in at least one pairwise comparison (false discovery rate [FDR] <0.1 and fold change [FC] ≥2).

We evaluated how well several recently published cell surface marker profiles, which define fibroblast subtypes largely based on tissue depth, tracked with the regional differences observed in our study (22). Among fluorescence activated cell sorting (FACS)-isolated, lineage-negative (29), Rainbow wound fibroblasts (Fig. 2*A* and *SI Appendix*, Fig. S3*A*), we found that most cells fell into the putative category of reticular fibroblasts (defined as DLK1^+^/SCA1^−^) rather than papillary (CD26^+^/SCA1^−^) or hypodermal (DLK1^+/−^/SCA1^+^) (*SI Appendix*, Fig. S3*B*). When we considered inner and outer wound fibroblasts separately, we found that distribution of fibroblast subtypes was not significantly different between these two groups (*SI Appendix*, Fig. S3*C*), suggesting that fibroblast subpopulations defined by selective marker profiles are not sufficient to delineate inner versus outer wound fibroblasts, though these can be readily distinguished based on their transcriptional programs even at the bulk tissue level.

### Single-Cell Transcriptomic Analysis of Injury Responsive Fibroblasts.

We sought to better characterize wound fibroblast heterogeneity by examining individual fibroblast transcriptional programs at important functional timepoints in the canonical wound healing process: POD 2, inflammation; POD 7, granulation; and POD 14, complete reepithelialization (“healed” wound). We conducted plate-based scRNA-seq of lineage-negative fibroblasts isolated based on their expression of Rainbow clone colors from both inner and outer wound regions at each timepoint (Fig. 2*A*). Four transcriptionally defined fibroblast subpopulations were identified (Fig. 2*B*), with considerable differences in their distributions between wound regions.

Given our interest in understanding lineage trajectories in the context of wound healing, we assessed the relative differentiation states of these fibroblast populations using CytoTRACE, a computational tool that leverages transcriptional diversity to order cells based on developmental potential (Fig. 2*C* and *SI Appendix*, Fig. S4) (17). This analysis identified a lineage trajectory stemming from scRNA-cluster 1, which is characterized by elevated expression of fibroblast markers such as *Pdgfra* and primarily represented by cells from the outer wound region, extending to scRNA-cluster 4, which is primarily represented by cells from the inner wound (Fig. 2*D*). These findings suggest that fibroblasts may undergo differentiation as they proliferate from the outer wound inward.

### Evaluation of Chromatin Accessibility Complements Transcriptional Analysis of Mechanoresponsive Fibroblast Subpopulations.

To evaluate the epigenomic changes associated with fibroblast activation and lineage differentiation in wound healing, we conducted a series of scATAC-seq experiments in parallel with our scRNA-seq assays (*SI Appendix*, Fig. S5 *A* and *B*). We identified considerable heterogeneity in accessibility profiles among individual wound fibroblasts, which were clustered into six epigenomically distinct subgroups using the ArchR platform (18) (*SI Appendix*, Figs. S5 *C* and *D* and S6 *A*–*D*). This partitioning was agnostic to the phenotype of cell origin (i.e., wound region or postoperative day), and all clusters included fibroblasts harvested from multiple timepoints and wound regions. We then performed cross-platform integration to link these scATAC data with our earlier scRNA data (18), resulting in four multimodal clusters characterized by both gene expression and chromatin accessibility profiles (Fig. 2 *E* and *F* and *SI Appendix*, Fig. S6*E*), which we refer to as ArchR-clusters 1 through 4.

We first examined the epigenomic landscape of the largest subpopulation, ArchR-cluster 1, which showed significantly elevated chromatin accessibility proximal to key fibrosis-related genes such as *Col1a1*, *Acta2*, and *Pdgfra* (Fig. 2*G* and *SI Appendix*, Figs. S7 *A* and *B* and S8), indicating that these cells are primed for their transcription. We also observed specific accessibility peaks and transcription factor footprinting in association with the FAK (*Ptk2*) locus and its downstream signaling elements such as Jun, suggesting that these fibroblasts may represent a mechanoresponsive, profibrotic subpopulation. ArchR-cluster 2 was associated with elevated *Fn1* and *Thbs1* accessibility peaks; ArchR-cluster 3 was characterized by increased accessibility at the *Jak2* locus and decreased accessibility at the *Fsp1* (*S100a4*) and *Il6st* loci; and ArchR-cluster 4 was characterized by increased accessibility at the *Ptk2b*, *Jak1*, and *Jak3* loci.

In addition to specific peak and motif evaluation, we also employed clusterwide enrichment analysis using the Genomics Regions Enrichment of Annotations Tool (GREAT) (30) (*SI Appendix*, Fig. S9*A*). We found significant enrichment for “increased fibroblast migration,” “focal adhesion,” and FAK-pathway signaling response elements in ArchR-cluster 1. Furthermore, pseudotime analysis of these integrated scRNA–ATAC data demonstrated an epigenomic progression from the putatively least-differentiated ArchR-cluster 1 to the remaining cell populations that was driven by mechanical signaling elements (*SI Appendix*, Fig. S9*B*).

Based on these findings, we provisionally characterized each subpopulation according to its putative role in the wound healing process: “mechanofibrotic” (ArchR-cluster 1), “activated-responder” (ArchR-cluster 2), “remodeling” (ArchR-cluster 3), and “proliferator” (ArchR-cluster 4) fibroblasts.

### Clonal Proliferation of Injury-Responsive Fibroblasts Is Mechanotransduction Dependent.

Our laboratory has previously shown that local tissue mechanics are crucial in guiding the response to healing after injury (31), and mechanotransduction signaling pathway elements were found to delineate fibroblast subpopulations in our scRNA and scATAC wound data. To further interrogate the role of local tissue mechanics in wound biology, we applied a small molecule FAK inhibitor (FAK_i_) to disrupt mechanosensation in stented mouse wounds (*SI Appendix*, Fig. S10*A*). Consistent with prior work, we found that FAK_i_-treated wounds healed at the same rate as untreated wounds (*SI Appendix*, Fig. S10 *B* and *C*) but resulted in significantly smaller and thinner scars composed of less-dense matrix tissue (*SI Appendix*, Fig. S10 *D* and *E*) (32).

To validate our FAK_i_ results, we conducted additional wound healing experiments using *Actin-Cre*^*ERT2*^*::Rosa26*^*VT2/GK3*^*::Ptk2*^*fl/+*^ and *Actin-Cre*^*ERT2*^*::Rosa26*^*VT2/GK3*^*::Ptk2*^*fl/fl*^ (heterozygous *Ptk2*^*fl/+*^] and homozygous [*Ptk2*^*fl/fl*^] knockout) mice, with local LiTMX induction at the time of wounding (*SI Appendix*, Fig. S10 *A*–*C*). We found that these mouse wounds also exhibited fewer scar-like patterns of connective tissue (*SI Appendix*, Fig. S10*E*). To further explore these differences, we employed an automated feature extraction algorithm (24) to quantify ultrastructure characteristics of wound tissue sections, which demonstrated that FAK_i_-treated wound specimens were more similar to unwounded skin than to vehicle-control wounds, including for both mature and immature collagen fiber intensities (*SI Appendix*, Fig. S10*F*). Taken together, these findings corroborate that when mechanotransduction is disrupted, wounds heal with thinner scars and connective tissue structure that is more similar to that of unwounded skin.

**Fig. 3.**
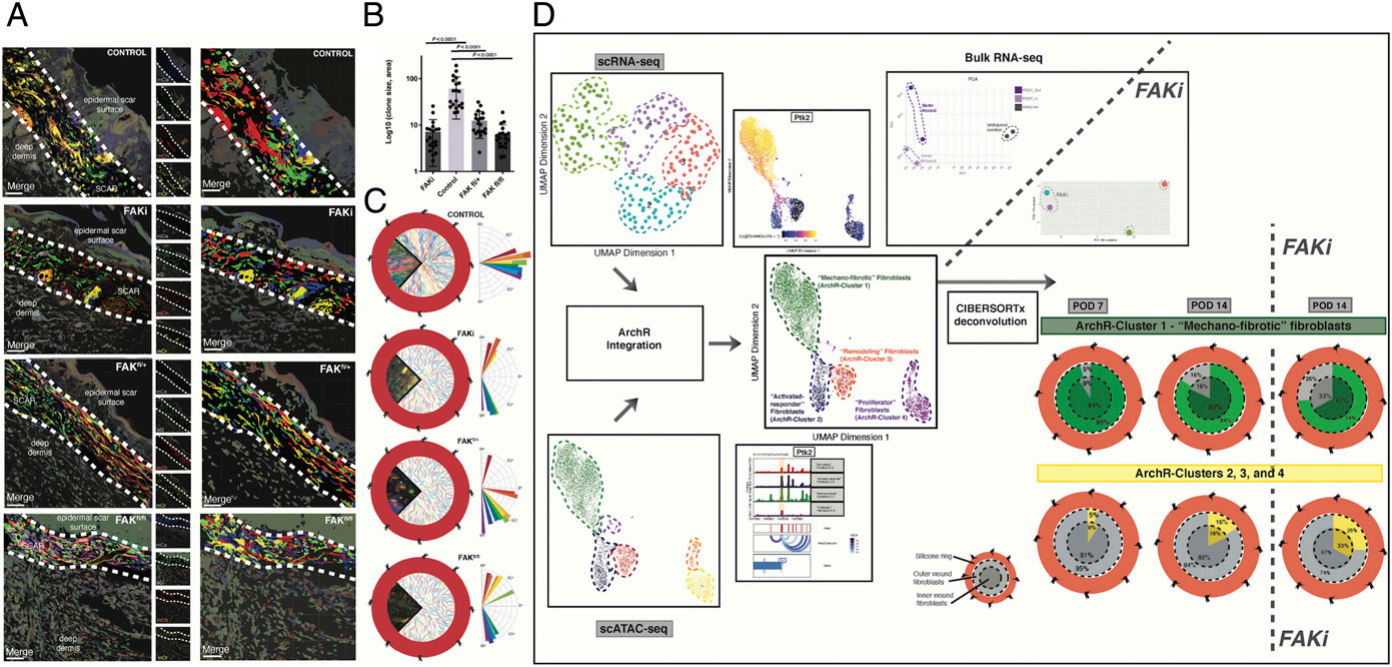
Clonal proliferation of injury-responsive fibroblasts is dependent on mechanotransduction signaling. (*A*) Representative confocal images of sectioned Rainbow mouse wound specimens treated with FAK_i_ (*Second*), FAK^fl/+^ (*Third*), or FAK^fl/fl^ (*Bottom*) compared with vehicle control (*Top*). Imaris rendering in second column of images highlights individual Rainbow clones. Dermal wound area highlighted with thick white dotted line. *n* = 5. (Scale bars, 25 μm.) (*B*) Quantitation of average clone size based on Imaris rendering. (*C*) Wedge sections of representative whole-mount confocal images of Rainbow wound specimens embedded within surrounding wound schematics for vehicle control (*Top*), FAK_i_-treated (*Second*), FAK^fl/+^ (*Third*), and FAK^fl/fl^ (*Bottom*) samples. Corresponding vector analyses are provided to the *Right* of each subpanel. (*D*) Schematic illustrating our approach to deconvolve bulk RNA-seq data using our multimodal scRNA–ATAC construct. Transcriptionally defined cluster labels from scRNA-seq analysis were projected onto the scATAC-seq manifold using an anchor transfer–based approach in ArchR as previously described (18) (*Left* column) to construct four multimodal fibroblast subpopulations. Putative names were assigned to these ArchR-clusters based on integrated functional and temporospatial characteristics. Feature and peak plots, above and below, for FAK (*Ptk2*) are provided for illustrative purposes (*Center* column). Deconvolution of bulk RNA-seq specimens representing wound fibroblasts treated with FAK_i_ versus vehicle control (*Right* column) was then performed using CIBRERSORTx (19) (*SI Appendix*, *Methods*). Wound schematics (with silicone ring around the outside, and outer and inner regions indicated) are provided to represent CIBRERSORTx output identifying changes in the percentages of ArchR-cluster 1 (mechanofibrotic) cells in bulk samples over time and with/without FAK_i_ treatment (shown in green). Parallel schematic of corresponding changes in other ArchR-clusters are provided in yellow.

To understand the transcriptional changes associated with modulation of mechanotransduction in wound healing, we conducted additional RNA-seq experiments comparing fibroblasts isolated from inner and outer regions of FAK_i_-treated and control wounds. We observed significant changes in the transcriptional programs of FAK_i_-treated cells and found that regional differences between inner and outer wound fibroblasts were dampened in wounds following FAK inhibition (*SI Appendix*, Fig. S11 *A* and *B*). These results suggest that local tissue mechanics contribute to transcriptional differences between inner and outer wound regions. We found that wound healing fibroblasts showed down-regulation of mechanotransduction- and fibrosis-related pathways with FAK_i_ treatment (*SI Appendix*, Fig. S11*C*). We also found that when mechanosignaling was blocked in Rainbow mice using FAK_i_, or in *Ptk2*^*fl/+*^ or *Ptk2*^*fl/fl*^ mice, the linear polyclonal proliferation of fibroblasts that was previously appreciated (Fig. 1*H*) was disrupted (Fig. 3 *A*–*C*), with smaller and less ordered Rainbow fibroblast clones.

We applied the deconvolution tool CIBERSORTx (19) to estimate the abundance of our four scRNA–ATAC populations (ArchR-clusters 1 through 4) within bulk RNA-seq data for fibroblasts isolated from POD 7 and POD 14 wounds with or without FAK_i_ treatment (Fig. 3*D*). We found that the majority of cell estimates across all specimens were attributed to mechanofibrotic ArchR-cluster 1, consistent with its prominent representation in both our scRNA-seq and scATAC-seq datasets. The predicted prevalence of these cells was highest at POD 7 and decreased by POD 14. FAK inhibition resulted in decreased representation of ArchR-cluster 1 fibroblasts at POD 14 for both inner and outer wound samples (compared to control wounds at POD 14), further supporting the mechanosensitivity of the putative mechanofibrotic ArchR-cluster 1 subpopulation.

### Spatial Transcriptomics Applied to Wound Healing.

**Fig. 4.**
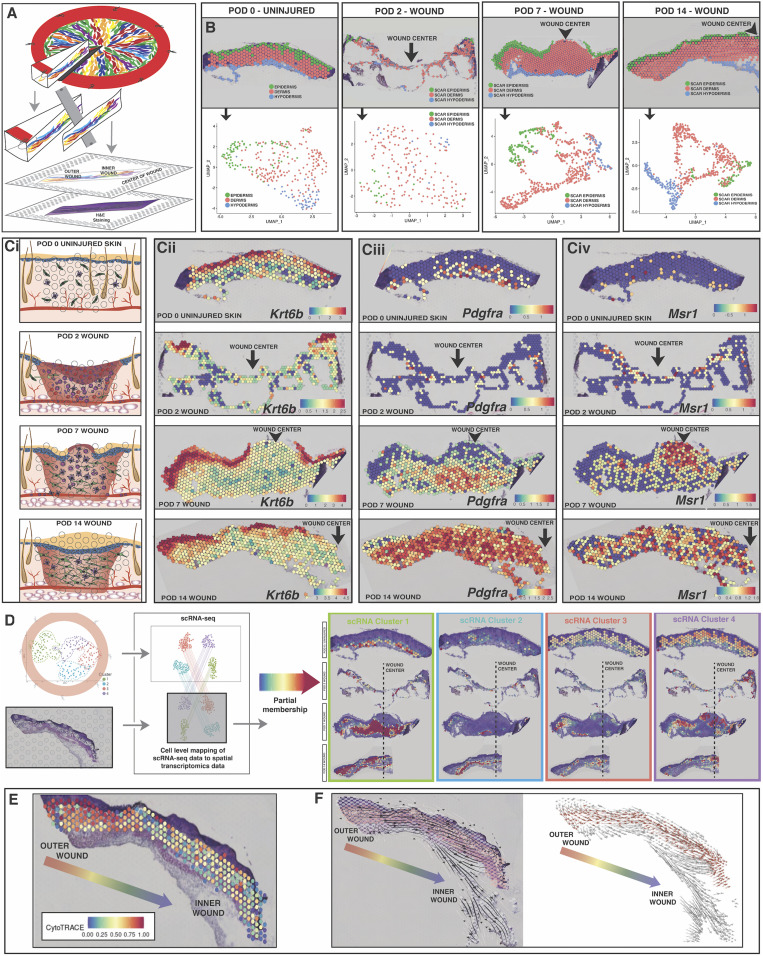
Spatial transcriptomics applied to wound healing and tracking of fibroblast subpopulations over time and space. (*A*) Schematic for generating spatial transcriptomics data from splinted excisional wounds using the 10× Genomics Visium protocol. Fresh Rainbow mouse wound tissue was harvested, flash frozen, embedded in optimal cutting temperature (OCT), and then sections were taken representing the complete wound radius. H&E staining and tissue section imaging were completed as described in the Visium protocol (*SI Appendix*, *Methods*). Each spot captures mRNA from 1 to 10 individual cells at that tissue location. (*B*) Delineation of scar layers based on underlying tissue histology at each timepoint (*Top* row), and UMAP plot showing that the three scar layers can easily be distinguished by their transcriptional programs, even independent of spatial information. (*C*) (*i*) Schematic of classic stages of wound healing evaluated at POD 2, 7, and 14 relative to uninjured skin. (*ii*) Keratinocyte activity as measured through expression of the *Krt6b* gene. (*iii*) Fibroblast activity as measured through expression of the *Pdgfra* gene. (*iv*) Immune cell activity as measured through expression of the *Msr1* gene. (*D*) Anchor-based integration of scRNA-seq populations (defined in Fig. 2*B*) with Visium gene expression to project partial membership within each spot across all timepoints. These populations exhibit strong spatial preferences within the wound.

To further explore the significance of fibroblast heterogeneity in healing wounds, we applied the recently developed 10× Genomics Visium platform to analyze gene expression while retaining tissue spatial information. We optimized and validated a protocol to enable highly reproducible Visium spatial transcriptomic analysis of skin and wounds across the healing process (*SI Appendix*, *Methods*). We then conducted spatial transcriptomic analysis on tissue from our stented Rainbow mouse wound healing model at POD 2, 7, and 14, as well as uninjured skin (Fig. 4*A*).

The epidermal, dermal, and hypodermal layers of the healing wounds were easily delineated histologically and also found to cluster independently based on transcriptional programs (Fig. 4*B*). Looking at individual genes for prominent wound healing cell types (Fig. 4 *C*, *i*), we found clear delineation of keratinocytes in the epidermis based on *Krt6b* expression (as well as other keratinocyte-specific genes), allowing us to examine reepithelialization over space and time at the transcriptional level (Fig. 4 *C*, *ii*). Similarly, fibroblast activity was evaluated using characteristic genes such as *Pdgfra*, which were most prominent in the dermis and most active at POD 14 (Fig. 4 *C*, *iii*). Likewise, by examining activated macrophage markers like *Msr1*, we could monitor these immune cells throughout our dataset and found that they were very prominent in the “proud flesh” at the center of the wound at POD 7 (Fig. 4 *C*, *iv*).

One challenge inherent in current spatial transcriptomic platforms such as Visium is that each “spot” (i.e., discrete spatial subregion from which transcripts are sequenced) can capture gene expression information from more than one cell (1 to 10 cells, characteristically). In a complex tissue such as a healing wound, this often includes cells of different types, particularly within the dermis where fibroblasts, multiple types of immune cells, and nascent blood vessels can be found. As such, to understand our spatial transcriptomics results in the context of our scRNA and scATAC fibroblast data, we needed to account for the contributions of nonfibroblast cells from each Visium spot. This was achieved by first estimating the number of each specific cell type present within individual spots based on the associated histological staining (*SI Appendix*, Figs. S12 *A*–*D* and S13). Cell counting was followed by random sampling in a Monte Carlo fashion to “subtract out” potential contributions from nonfibroblast cells, generating a distribution of 10,000 inferred fibroblast transcriptomes for each Visium spot. These were propagated forward for anchor-based integration to generate and pool spatially overlaid partial memberships for each of our four scRNA-clusters (Fig. 4*D*).

We found that the predicted spatial distributions for our scRNA-seq clusters were largely congruent with the transcriptional differences observed earlier between inner and outer cells using our microdissection approach (e.g., fibroblasts belonging to the mechanofibrotic cluster became more prominent over time, expanding from the outer to inner wound regions to fill the scar). Upon further examining transcriptional programming relative to tissue depth, we observed clear spatial distinctions between the apical and basal regions of the dermis as early as POD 7 and most prominently at POD 14 (*SI Appendix*, Figs. S14 *A*–*F* and S15 *A* and *B*). For example, the MMP inhibitor *Timp1* is expressed by fibroblasts in the basal dermis, while *Thbs2*, which mediates cell–matrix interactions, is primarily expressed in the more apical scar region.

To assess the relative differentiation states of fibroblasts in this system, we applied CytoTRACE to our POD 14 dermal scar data and found that, similar to our RNA-seq microdissection findings, fibroblasts exhibited significantly less transcriptional diversity in inner wound regions, further supporting fibroblast differentiation from the outer to the inner wound regions during tissue repair (*SI Appendix*, Fig. S16).

### Integrated Analysis Permits Imputation of Spatial Epigenomic Properties.

**Fig. 5.**
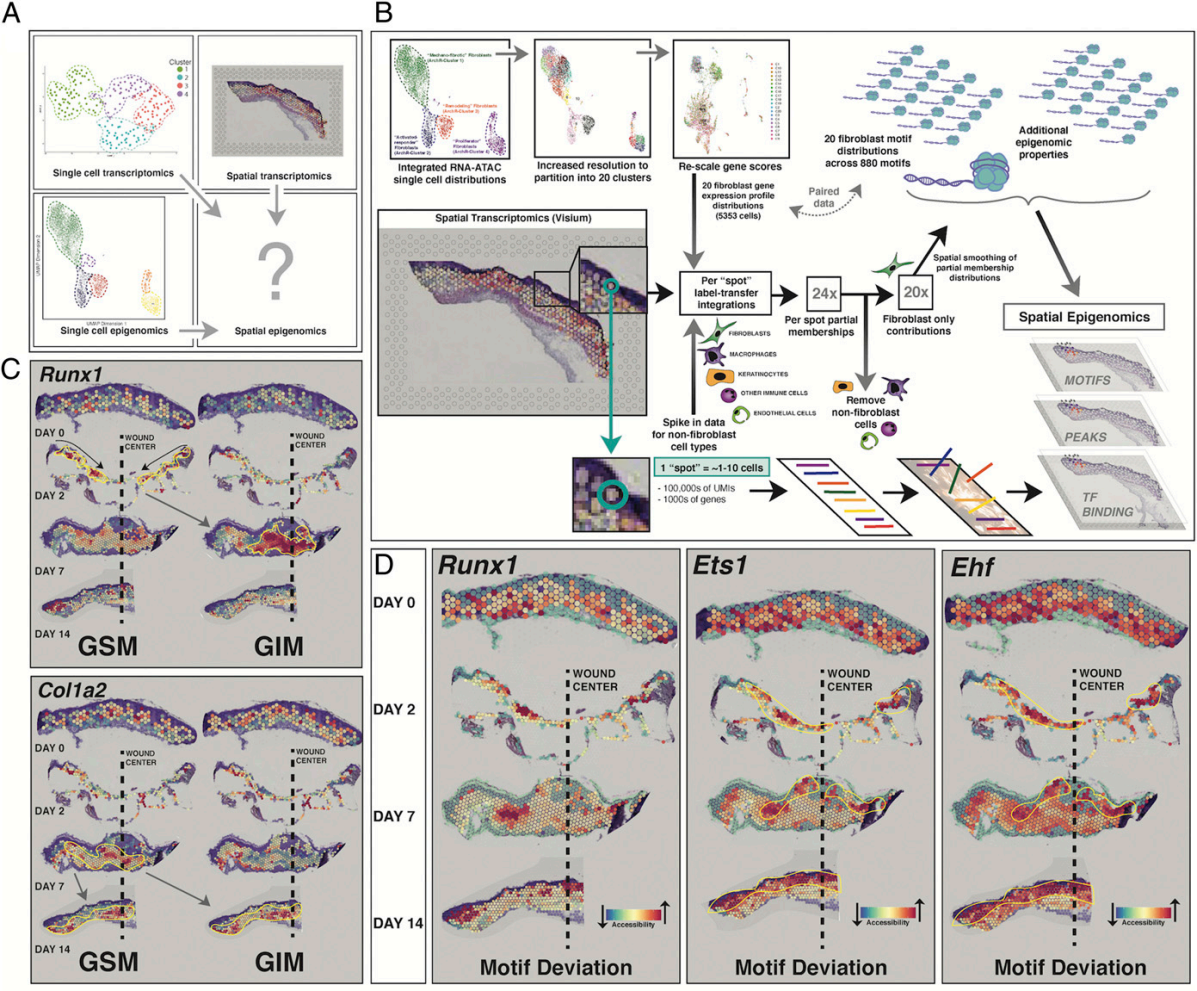
Integrated analysis permits imputation of spatial epigenomic properties. (*A*) Punnett square schematic summarizing the data acquired in Figs. 2 and 4; setting the stage for imputation of spatial epigenomics. (*B*) Schematic summarizing imputation of spatial epigenomics. Multimodal scRNA–ATAC fibroblast data were first reclustered into a higher-resolution space to generate 20 partitions, each representing between 27 and 552 cell equivalents. Gene score matrix distributions, informed by both modalities, were then extracted for each partition and subjected to SCT transformation. “Spike-in” RNA-seq data for keratinocytes, endothelial cells, granulocytes, and macrophages were obtained from pure Visium spots across all timepoints. These data were combined and subjected to a similar variance-stabilizing transformation. The resulting putative single-cell gene expression reference matrix was then used to assign initial partial set memberships for each spatial transcriptomic datapoint using an anchor transfer–based approach. Nonfibroblast contributions were subsequently regressed out, and a single-step spatial smoothing filter was applied to the resulting membership space, followed by renormalization. The resulting partial set memberships for each spatial datapoint were then treated as a topological vector space, onto which epigenomic peak, motif, and binding activity from the 20 scRNA–ATAC partitions can be projected. (*C*) Visium plots showing POD 0, 2, 7, and 14 (*Top* to *Bottom*) wound sections, imputed spatial epigenomics. For housekeeping genes such as *Hprt* (*Top*), gene imputed matrix (GIM) correlates with gene score matrix (GSM) epigenomic data and is fairly stable over space and time (*Top*). However, for *Runx1*, which we have shown to be very active within wound fibroblasts, GSM data show opening at the *Runx1* motif at POD 2, which yields strong gene expression primarily among inner wound fibroblasts at POD 7 (*Bottom*). (*D*) Visium plots showing POD 0, 2, 7, and 14 (*Top* to *Bottom*) wound sections, motif deviations for genes of interest related to FAK-mediated mechanotransduction, and fibroblast proliferation including *Runx1*, *Ets1*, and *Ehf.*

To further explore fibroblast cell fate with spatial resolution, we developed a method to combine our integrated single cell RNA–ATAC framework with Visium in order to impute spatially informed epigenomes for wound healing fibroblasts (Fig. 5*A*). As described above, we generated spatial transcriptomic data from unwounded skin and POD 2, 7, and 14 wounds. To extend this analysis to impute spatial epigenomic properties, we used our RNA–ATAC construct to ascribe partial membership values to fibroblasts present within each Visium spot. This was achieved by first subtracting out putative nonfibroblast contributions as described above, followed by anchor-based mapping into a higher-dimensional cluster space from our gene integration matrix (Fig. 5*B* and *SI Appendix*, Table S1). Parameterization was optimized to preserve spatial autocorrelation for the top measured and imputed gene expression distributions within the POD 14 dermis (*SI Appendix*, Fig. S17 *A* and *B*). To account for residual contributions from nonfibroblast cells that may remain after our initial subtraction step, we also spiked in RNA-seq data for keratinocytes, endothelial cells, macrophages, and neutrophils. The resulting putative reference matrix was then used to assign initial partial set memberships for each spatial datapoint using an anchor transfer–based approach. A single-step spatial smoothing filter was applied to this membership space, followed by removal of nonfibroblast contributions and renormalization. The resulting partial set memberships for each spatial datapoint then allowed us to project higher-order epigenomic features from the scRNA–ATAC data onto these Visium samples (*SI Appendix*, Fig. S18 *A*–*D*). These spatial epigenomic imputations provided a valuable complement to further refine our understanding of the fibroblast biology driving tissue repair. Detailed data analysis is provided in Fig. 5 *C* and *D* and *SI Appendix*, Figs. S19 and S20 and more broadly summarized below for each timepoint in the healing process.

Immediately following wound injury, tissue trauma leads to inflammatory cell recruitment, provisional clot formation, and a dermal gap resulting in loss of contact inhibition among local fibroblasts. These fibroblasts are recruited into the wound bed and begin proliferating. Our data suggest that by POD 2, subsets of these cells have differentiated along the wound margin to form a putative Activated-Responder Fibroblast subpopulation. Other, less-differentiated and more mechanosensitive (mechanofibrotic), fibroblasts become preactivated in the deeper dermis at this point, increasing chromatin accessibility for *Runx1*, which is a primary regulator of mesenchymal progenitor cell proliferation and differentiation (33).

By POD 7, macrophage-dominated granulation tissue occupies the central wound defect, allowing overlying keratinocyte proliferation and reepithelialization. At this time, mechanofibrotic Fibroblasts begin to differentiate as they finish migrating toward the wound center, where they appear to transition to a more Proliferator subpopulation. These cells are strongly profibrotic and characterized by elevated *Spp1* gene expression and chromatin accessibility. In parallel, a population of Remodeling Fibroblasts begins to appear in the outer deep dermis (Fig. 4 *C* and *D* and *SI Appendix*, Fig. S15 *A* and *B*).

At POD 14, reepithelialization is complete, and the wound is traditionally considered to be healed. However, while keratinocyte activity does decrease at this time (consistent with completion of reepithelialization), there remains a strong immune cell presence, supported by continued wound fibroblast chemokine secretion, to stimulate active fibrosis in the dermal layer (*SI Appendix*, Fig. S14 *E* and *F*).

Considering our imputed spatial epigenomics data more globally, we observed that changes to chromatin accessibility frequently preceded downstream changes in gene expression, even within the constraints of our coarse temporal sampling (Fig. 5 *C* and *D* and *SI Appendix*, Fig. S19 *A*–*C*). For example, we found that the *Runx1* motif, which is downstream from and regulated by FAK mechanotransduction, initially becomes open at POD 2, remains open particularly along the leading wound edge at POD 7, and then begins to decrease in accessibility throughout the nascent scar at POD 14. Similarly, *Col1a2* motif opening precedes a dramatic increase in *Col1a2* gene expression seen in the POD 14 wound scar.

In aggregate, these studies represent a framework for the comprehensive elucidation of wound healing fibroblast phenotypes based on both gene expression and chromatin accessibility across time, space, and lineage. Furthermore, these findings allow us to reevaluate the classical stages of wound healing, typically described as three overlapping phases: inflammation (POD 2), proliferation (POD 7), and remodeling (POD 14) (3). Based on our findings, we propose reframing these overlapping stages as: 1) Early inflammation, in which immune cells are migrating and infiltrating the injury site without proliferation; 2) reepithelialization, which includes rapid keratinocyte proliferation across the wound surface, fibroblast recruitment, and macrophage proliferation; and 3) activated fibrosis, where maximal fibroblast activation is achieved and sustained in a slow asymptotic decay by steady-state inflammatory signaling beneath the healed wound (*SI Appendix*, Figs. S21 *A*–*D* and S22 *A* and *B*).

## Discussion

In this manuscript, we define fibroblast biology throughout the course of wound healing using integrated, single-cell multimodal -omics to unravel the spatial, temporal, and functional heterogeneity of these cells. We demonstrate that fibroblasts are activated from tissue-resident cells in response to injury and proliferate polyclonally to fill the wound gap. Furthermore, we demonstrate that fibroblasts undergo spatially informed differentiation during this process.

Elucidating these relationships required the integration of nascent technologies and data platforms in what is still a rapidly evolving field of multiomic imputation. This work demonstrates the paired analysis of single-cell RNA and chromatin accessibility with spatial resolution in the context of tissue repair. This approach provides a unique lens through which we can view complex cell processes, and specifically allowed us to demonstrate that upstream chromatin changes surrounding mechanical signaling elements precede transcriptional activation and cell proliferation, thus suggesting a mechanistic link from tissue force to activation of wound healing fibroblasts.

Furthermore, we were able to identify and characterize putative, functionally distinct fibroblast subpopulations with divergent transcriptional and epigenomic programs. We provisionally designate these four wound healing fibroblast phenotypes as Mechanofibrotic, Activated Responder, Proliferator, and Remodeling. Following skin injury, fibroblasts are locally recruited and migrate to the wound. By POD 2, a subset of fibroblasts appears to have differentiated to form an activated-responder subpopulation, while the remaining outer wound fibroblasts comprise the less differentiated mechanofibrotic cells. The latter fibroblasts highly express known fibrosis-associated markers such as *Engrailed-1* (23, 24), *Col1a1* (34), *Tgbf2* (35), and *Jun* (36). At POD 7, mechanofibrotic cells begin to differentiate in response to mechanotransduction cues as they migrate toward the wound center. By POD 14, despite complete epithelialization, healed wounds remain in a steady state of fibrosis, maintained through sustained inflammatory signaling within scar tissue. Additional studies examining even later timepoints will be required to further characterize the dynamics of these cells within the healed scar tissue.

Taken together, these results illustrate fundamental principles underlying the cellular response to tissue injury. We demonstrate that populations of fibroblasts migrate, proliferate, and differentiate in an adaptive, dynamic response to disruption of their local mechanical environment. Understanding the origin, activation, and differentiation trajectories of injury-responsive cells is critical to develop therapeutic strategies to promote optimal tissue repair.

## Materials and Methods

All animal experiments were approved by and conducted in accordance with the regulations of the Stanford University Animal Care and Use Committee. Mouse husbandry, transgenic models, immunostaining, high-throughput -omics, and computational methods are described in *SI Appendix*.

## Supplementary Material

Supplementary File

## Data Availability

All sequencing datasets generated in this study are freely available through the Gene Expression Omnibus (GEO), with accession number GSE178758.
