# Supplementary material for: Integrated spatial multiomics reveals fibroblast fate during tissue repair
Source: Proc Natl Acad Sci U S A. 2021 Oct 7;118(41):e2110025118. doi: 10.1073/pnas.2110025118 (PMC8521719; doi:10.1073/pnas.2110025118)
Supplement: Supplementary File [file pnas.2110025118.sapp.pdf]

Integrated spatial multi-omics reveals fibroblast fate during tissue repair

Supplemental Information

METHODS

Animal Models

The following mouse strains were purchased from Jackson Laboratories: Black/6 (C57BL/6J), Actin-Cre^{ERT2} mice (Tg(CAG-cre/Esr1)5Amc/J), eGFP (C57BL/6J-Tg(CAG-EGFP)10sb/J), and FAK^{flox} (B6.129P2(FVB)-*Ptk2*$^{tm1.1Guan}$/J). αSMA-Cre^{ERT2} were courtesy of Dr. Ivo Kalajzic, University of Connecticut. Rainbow mice (ROSA26$^{VT2/GK3}$) were courtesy of the Weissman Laboratory, Stanford University School of Medicine. All of the mice were genotyped as per manufacturer's recommendations. Female mice were used for all experiments in this study. Mice were housed at the Stanford University Comparative Medicine Pavilion (CMP) and Research Animal Facility (RAF). The facilities provided light- & temperature-regulated housing. Mice were given rodent chow and water *ad libitum*. A minimum sample size of three animals was used for all experiments (exact numbers for experiments are noted in the figure legends). Animals with appropriate genotypes for a given experiment were randomly allocated to the various experimental conditions. All experiments were completed according to the Stanford University Animal Care and Use Committee standards of care.

Splinted Excisional Mouse Wound Healing Model

Splinted excisional wounds were created following the protocol outlined by Galiano *et al (1),* which was designed to mimic human wound healing kinetics. In brief, mice were anesthetized with isofluorane (Henry Schein Animal Health) at a concentration of 1-2% in oxygen at 3 L/min. The mice were placed in prone position, and dorsal fur was removed. Skin was sterilized with a betadyne wash followed by 70% ethanol. Punch biopsies were used to make 6mm diameter full-thickness dermal wounds. Two dorsal wounds were created on each mouse. A silicone ring was fixed to the dorsal mouse skin using an adhesive and interrupted 6-0 nylon sutures placed around the outer edge of the ring to prevent rapid contraction. A sterile dressing was placed and changed

every other day until the wound was harvested. Digital photographs were taken at the time of surgery and every other day at the time of dressing changes.

Parabiosis

Parabiotic mouse pairs were created as previously described (2). In brief, parabiotic pairs consisted of one female wild-type (C57BL/6) mouse and one female eGFP (C57BL/6-Tg(CAG^{EGFP})10sb/J) mouse that were both age-matched and housed together for 2 weeks prior to parabiosis surgery. Mice were anesthetized, shaved, and sterilized as previously described. Matching incisions were made from the base of the elbow joint (olecranon) to the base of the knee joint on corresponding sides of each mouse. The joints and skin were sutured together. Peripheral blood chimerism was determined with flow cytometry two weeks after parabiosis surgery. Wound surgery was performed on the wild-type mouse after systemic circulation was established.

Liposomal Tamoxifen Induction

Liposomal tamoxifen (LiTMX) was created as per the protocol described by Ransom et al(3). Briefly, liposomes were applied locally (pulse) to the surface of dorsal mouse wounds to induce Cre recombinase at the time of wounding. Wound surgeries were conducted as described above.

Tissue Processing

Mouse tissue was fixed in 4% paraformeladehyde (Electron Microscopy Sciences) for 20 hours at 4°C and embedded into paraffin per standard protocols. For cryopreservation, following fixation, specimens were placed in 30% sucrose (Sigma) at 4°C until saturation, followed by OCT at 4°C until saturation and embedded in OCT. Representative tissue specimens were sectioned and stained with hematoxylin and eosin (H&E, Sigma-Aldrich), Picro Sirius Red Stain (Abcam), or Masson’s trichrome (Sigma-Aldrich) per the manufacturer’s protocols.

Whole Mount

A border of Vaseline was prepared on a Superfrost/Plus microscope slide and the center was filled with mounting medium (BABB clearing reagent or fluoromount-G (SouthernBiotech)). The tissue sample was placed into the reservoir of mounting medium and a cover slip was applied. The whole-mounted samples were stored at 4°C.

Tissue Clearing

Tissue clearing optimized to preserve expression of endogenous fluorophores as previously described by our laboratory(4) was pursued on selected Rainbow whole mount and sectioned wound specimens. In brief, for dehydration, tert-butanol (FisherSci) was buffered to a pH 9.5 with triethylamine (FisherSci). Fixed tissue specimens were placed into increasing gradients of tert-butanol (33%, 66%, and 100%) at room temperature for 30 minutes each and then left in 100% tert-butanol overnight. Tert-butanol and benzoic acid:benzyl benzoate (Sigma Aldrich) at a 1:2 ratio were buffered to pH 9.5 with triethylamine (FisherSci). For whole mount samples, tissues were placed in the prepared BABB solution for clearing for 7 hours at room temperature. Cleared whole mount and sectioned tissue specimens were stored in BABB solution at 4°C.

Confocal Imaging and Analysis

Rainbow mouse tissues were fixed and prepared in the dark to minimize bleaching of endogenous fluorophore expression. Laser scanning confocal microscopy of whole mount and tissue specimens was performed using the Leica WLL TCS SP8 Confocal Laser Scanning Miscroscope (Leica Microsystems) located in the Cell Sciences Imaging Facility (Stanford University, Stanford, CA). Both the 20x and 40x objectives were used for imaging (x20 and x40 HC PL APO IMM CORR CS2, H_2O/glycerol/oil, numerical aperture 0.75). Precise excitation and hybrid detection of the Rainbow fluorophores (mCerulean, eGFP, mOrange, and mCherry) was captured. Raw image

stacks were imported into either Fiji (NIH) or Imaris (Bitplane/Perkin Elmer) software for further analysis. Z-stacked confocal images were rendered into 3-dimensions. Analysis of clonal volume, area and direction was conducted using the surface and thresholding tools using Imaris software.

Immunostaining

Cryosections on Superfrost Plus microscope slides (FisherSci) were rehydrated and permeabilized with 0.5% Triton X-100 (Sigma). Tissue sections were then rinsed repeatedly, incubated with 1X Power Block (BioGeneX), and stained with primary antibody for one hour at room temperature. Primary antibodies used for immunostaining included CD45 (D3F8Q) Rabbit mAb (Cell Signaling), Rabbit mAb to alpha Smooth Muscle Actin (Abcam), Rb pAb to Collagen I (Abcam), Rb pAb to Collagen III (Abcam), Rb pAb to Collagen III (Abcam), Specimens were then rinsed repeatedly, stained with a secondary antibody for one hour, rinsed repeatedly, and mounted with ProLong Gold antifade reagent with DAPI. Secondary antibodies used for immunostaining included Goat anti-Rabbit Secondary Antibody Alexa Fluor 488 (Abcam), Goat anti-Rabbit Secondary Antibody Alexa Fluor 647 (Abcam), Donkey Anti-Rat Secondary Antibody Alexa Fluor 647 (Abcam), and Donkey Anti-Rabbit Secondary Antibody Alexa Fluor 555 (Invitrogen). Slides were then coverslipped and imaged using a Lecia DMI6000B inverted microscope or confocal microscopy as described above.

DAB Immunohistochemistry

Cryosections on Superfrost Plus microscope slides (FisherSci) were rehydrated. H2O2 was used to quench endogenous peroxidases. Antigen retrieval was conducted using Abcam's Trypsin Antigen Retrieval kit (Ab970) per the manufacturer's protocol. Tissue sections were then permeabilized with 0.025% Triton X-100 (Sigma), rinsed, incubated with 1X Power Block (BioGeneX), and stained with primary antibody at 4°C overnight. Primary antibodies used for staining included Phospho-FAK/PTK2 pTyr397 (Invitrogen) and Il6 Monoclonal Antibody (MP5

20F3) (Invitrogen). Tissue sections were then washed, and incubated with secondary antibody at room temperature for one hour. Secondary antibodies used for staining included Horse anti-Rabbit IgG Antibody (H+L) Biotinylated R.T.U. (Vector Laboratories) and Goat anti-Rat IgG Antibody (H+L) Biotinylated R.T.U. (Vector Laboratories). The VECTASTAIN Elite ABC system for avidin/biotin peroxidase (Vector Laboratories) was then applied per the manufacturer's recommendations. DAB staining was conducted using the BD DAB Substrate Kit (BD 550880) per the manufacturer's guidelines. Specimens were co-stained with Hematoxylin, rinsed, dehydrated, mounted, and imaged using a brightfield microscope.

Automated Connective Tissue Analysis

Polarization microscopy images of Picrosirius Red-stained histology specimens were obtained at 40X with a minimum of 10 images per specimen. Images were color deconvoluted to yield separated images of mature (red) and immature (green) connective tissue fibers. These images were then separately denoised, binarized, and quantified across a panel of fiber parameters (length, width, persistence, alignment, etc.), for a total of 294 parameters per image (147 for mature fibers, 147 for immature fibers). Images were quantitatively compared by t-SNE, an algorithm for visualizing high dimensionality data in two dimensions. Individual parameter values were compared between specimen conditions.

Sample Preparation and Fluorescent-Activated Cell Sorting (FACS) Isolation

Mouse wound tissues were harvested and micro-dissected to separate the outer and inner regions of the wounds. In brief, the wound radius was divided in half, and microdissection was conducted to separate the inner radius and the outer ring based on this division. The wound tissue regions were then placed independently in a dispase-trypsin solution as previously described (5) for 30 minutes at 37ºC; following this, the epidermis and hypodermis were isolated from the dermal wound scar specimens and discarded. The dermal wound tissues were then minced and

digested in DMEM-F12 (GIBCO®) with 0.5mg/ml Liberase™ (Roche) for 30 minutes at 37º in an orbital shaker. The digests were quenched with quench media (DMEM-F12 with 10% Fetal Bovie Serum (FBS, Sigma)), centrifuged at 300 x G for 5 minutes at 4°C, resuspended in quench media, filtered through 100, 70, and 40μm cell strainers (Falcon cell strainer, ThermoFisher), centrifuged once more, and resuspended in FACS buffer.

Cells were counted. Primary antibodies were then applied, and cells were stained in the dark on ice with gentle agitation for 30 minutes. Antibodies against the following cell surface markers primarily or secondarily conjugated to the same fluorophore were used for exclusion of "lineage" cells in order to isolate fibroblasts in an unbiased manner: CD45, CD31, Ter119, Tie2, CD324, and CD326. This approach has been previously validated by our laboratory to isolate mouse wound fibroblasts(6, 7). Cells were then washed with FACS buffer, centrifuged, and resuspended in FACS buffer. Staining with any secondary antibodies was conducted in the same manner. SYTOX ADvanced Ready Flow Reagent (ThermoFisher) or DAPI (Thermofisher) were used as viability markers. Fibroblasts were isolated using the FACS Aria II system.

For bulk RNA-seq, cells were sorted into chilled lysing reagent under RNA/DNAse-free conditions (Trizol LS, ThermoFisher). For scRNA-seq, cells were bulk sorted by "purity" based on expression of Rainbow colors. These sorted cell aliquots were then individually re-sorted by single cell and index into prepared, 96-well plates containing lysis-buffer. For scATAC-seq, cells were sorted into FACS buffer. mCerulean was arbitrarily selected from among the available Rainbow colors and mCerulean+ wound fibroblasts were used for single-cell sequencing experiments. Flow-cytometry plots shown are representative of at least three independent experiments.

For flow cytometry analysis of cell surface and phosphorylated proteins, a single cell suspension was prepared using manual tissue dispersion rather than enzymatic digestion to preserve phosphorylated signal. Rainbow fibroblasts were isolated as described above and then prepared using the BD Biosciences Cytofix/Cytoperm™ kit according to manufacturer's

instructions. Primary antibodies used for flow cytometric analysis included Phospho-FAK/PTK2 pTyr397 (Invitrogen), DLK1 Monoclonal Antibody (3A10) (Invitrogen), Rat Anti-Mouse Sca1 (Ly-6A/E) (Stem Cell technologies), Rabbit Anti-DPP4 (CD26) (Abcam). Protein expression analysis was conducted using the FACS Aria II system.

FACS gating and data analysis was performed using FlowJo. Gating schemes were established with fluorescence-minus-one controls. Single cells were first gated using FSC and SSC parameters. Dead and lineage-positive (non-fibroblast) cells were then excluded. Gating schemes to quantitate and/or isolate fibroblasts were validated by plating a portion of the sorted cells for morphological visualization.

Bulk mRNA Sequencing

RNA extraction was performed using Qiagen miRNeasy kit with on column DAnase treatment per the manufacturer's recommendations. The Clontech Smarter Ultra Low Input RNA kit (Takara Bio) was used to generate cDNA from 150 pg total RNA following the manufacturer's recommendations. Amplified cDNA was purified using SPRI Ampure Beads (Beckman Coulter) and the quality and quantity was measured using a High Sensitivity DNA chip on the Agilent 2100 Bioanalyzer (Agilent Technologies). cDNA was sheared to an average length of 300 basepairs using a Covaris S2 ultrasonicator (Covaris) and libraries were generated with the Clontech Low Input Library Prep kit (Takara Bio). The samples were uniquely barcoded, pooled, and sequenced on HiSeq (Illumina).

Bulk mRNA Sequencing Data Analysis

A total of 12 mouse samples were profiled by bulk RNA-sequencing as described above. Raw FASTQ reads were aligned to the GENCODE vM20 reference transcripts (GRCm38.p6) with Salmon(8) v0.12.0 using the --seqBias, --gcBias, --posBias, --useVBOpt, --rangeFactorizationBins 4, and --validateMappings flags and otherwise default parameters for

single-end mapping. Salmon results were merged into a single gene-level counts matrix using the R package, tximport(9) v1.4.0. Count normalization and differential gene expression analysis was performed using the DESeq2 v1.22.2 package in R or using Basepair software (www.basepairtech.com). Counts were size-factor normalized using the DESeq function and log2-transformed. Pairwise differential gene expression analysis was performed using the lfcShrink function and indicating type = apeglm, which applies the adaptive t prior shrinkage estimator. As recommended, a threshold of *P*-adjusted < 0.1 was used to define significance for differentially expressed genes.

Single-cell RNA Sequencing (scRNA-seq)

Mouse wound healing fibroblasts were prepared for PlateSEQ analysis as described above. In brief, mouse dermal scar specimens derived from 4 litter mates were pooled for each timepoint (POD 2, 7, and 14) and divided by wound region (inner and outer). Cells were counted and filtered just prior to loading into the FACS machine. Fibroblasts were FACS-isolated based on expression of individual Rainbow colors, and using an unbiased, lineage-based strategy to isolate fibroblasts independent of cell surface marker expression(7). The sorted mCerulean+ (arbitrarily selected from among the available Rainbow colors) fibroblast aliquot was then re-, index-sorted into 96-well plates with 4 ul per well of lysis buffer consisting of 1U/ul of Recombinant RNase inhibitor (RRI) (Clontech), 0.1% Triton X-100 (Thermo 85111), 2.5mM dNTP (ThermoFisher), 2.5 uM oligodT30VN (5′AAGCAGTGGTATCAACGCAGAGTACT30VN-3′, IDT). Once sorted, plates were immediately spun down and frozen at -80°C. Single cell RNAseq was performed via the method described by Picelli et al(10). Lysis buffer plates were thawed on ice, then heated at 72 ^{o}C/3 min in a Biorad C1000 Touch thermal cycler to denature RNA. First strand cDNA synthesis was performed in a 10 ul reaction with 100 Units of Clontech's Smartscribe reverse transcriptase (Cat. No. 639538), 10 Units RRI, 1X First Strand Buffer (Clontech), 5 mM DTT, 1M Betaine (Sigma B0300-5VL), 6mM $MgCl_2$, 1 μM Template Switch Oligo (TSO, (5′-

AAGCAGTGGTATCAACGCAGAGTACATrGrG+G-3′, Exiqon) at 42°C/90 min, 70°C/5 min. PCR pre-amplification was performed in a 25 ul reaction with 1X Kapa HiFi HotStart, 0.1 uM ISPCR primer (5′-AAGCAGTGGTATCAACGCAGAGT-3′, IDT) at 98°C/3 min, then 25 cycles of 98°C/20 sec, 67 °C/15 sec, 72 °C/6 min, then 72 °C/5 min. Amplified cDNAs were purified by SPRI beads using a Biomek FX automated platform and eluted in 25 ul water, and 2 ul aliquots were run on an Fragment Analyzer High Sensitivity NGS 1-6000 kit for quantitation. Barcoded sequencing libraries were made using the miniaturized Nextera XT protocol of Mora-Castilla et al(11) in a total volume of 4 ul. Pooled libraries were analyzed on an Agilent Bioanalyzer High Sensitivity DNA chip for qualitative control purposes. cDNA libraries were sequenced on a NextSeq 500 Illumina platform aiming for 50,000 reads per cell.

scRNA-seq Data Processing

Fastq files for individual cells were converted to BAM format using STAR v2.5.3. Cell barcodes representative of quality cells were delineated from barcodes of apoptotic cells or background RNA based on a threshold of having at least 200 unique transcripts profiled and less than 10% of their transcriptome of mitochondrial origin, resulting in 191 unique single cells, with an average of 550,000 reads per cell. Raw mRNA counts were normalized on a per-cell basis with a scale factor of 10,000 and subsequently natural log transformed with a pseudocount of 1 in R (version 3.6.0) using the Seurat package (version 3.1.1)(12). Aggregated data was then evaluated using uniform manifold approximation and projection (UMAP) analysis over the first 15 principal components(13), with n.neighbors = 50, min.dist = 0.75, and repulsion.strength = 1.

Generation of Characteristic Subpopulation Markers and Enrichment Analysis

Cell-type marker lists were generated with two separate approaches. In the first approach, we employed Seurat's native *FindMarkers* function with a log fold change threshold of 0.25 using the ROC test to assign predictive power to each gene. However, in order to better account for the

mutual information contained within highly correlated predictive genes, we also employed a characteristic direction analysis (14). The 50 most highly ranked genes from this analysis for each cluster were used to perform gene set enrichment analysis in a programmatic fashion using EnrichR (version 2.1) (15).

Prediction of Differentiation States

CytoTRACE v0.2.1 R package (publicly available at https://cytotrace.stanford.edu)(16) was used with default settings to predict differentiation states in scRNA-seq data. Predictions were generated using the CytoTRACE function with a read count matrix as input. Low-dimensional plots for visualizing CytoTRACE and cluster assignments were generated with plotCytoTRACE using UMAP coordinates generated from Seurat. Genes associated with less and more differentiated cells were generated with plotCytoGenes.

Single-cell ATAC Sequencing (scATAC-seq)

Single cell ATAC-seq was performed following 10x Genomics protocols. In brief, Rainbow mouse wound healing fibroblasts were FACS-isolated using the unbiased, fibroblast-isolation strategy as described above. As for scRNA-seq, mouse dermal scar specimens derived from 4 litter mates were pooled for each timepoint (POD 2, 7, and 14) and divided by wound region (inner and outer). As for scRNA-seq, the sorted mCerulean+ (arbitrarily selected from among the available Rainbow colors) fibroblast aliquot was selected for further analysis. For nuclei isolation, 10x genomics protocol CG000169 Rev D was followed using the low cell input modifications. Transposition, GEM generation and barcoding, post GEM Incubation Cleanup, library construction, and quantification was conducted following the 10x genomics protocol CG000169 Rev D. Pooled libraries were analyzed on an Agilent Bioanalyzer High Sensitivity DNA chip for qualitative control purposes. cDNA libraries were sequenced on a NextSeq 500 Illumina platform. In total, we

generated scATAC-seq profiles from 5,353 cells, which yielded on average 13.0 × 10^3 unique fragments mapping to the nuclear genome.

scATAC-seq Data Processing and Analysis

Raw base call (BCL) files were demultiplexed to fastq files using the 10x Genomics Cell Ranger tool *cellranger-atac mkfastq*. These files were then aligned to the mouse genome (mm10) using *cellranger-atac count* with default parameters. Downstream analysis of scATAC-seq data were performed using ArchR, a novel tool developed by our collaborators (Granja et al., 2020). Arrow files were created for each sample using TSS and frag filters of 4 and 1000, respectively. Doublets were filtered using k = 10 pseudodoublets embedded in UMAP space, as previously described (Granja et al., 2020). Dimensionality reduction was achived using ArchR's implementation of latent semantic indexing (LSI). Initial clustering was achieved using the native *addClusters* function with resolution = 0.1, method = Seurat, reducedDims = IterativeLSI. Single cell embeddings were then generated using *addUMAP* with nNeighbors = 40, minDist = 0.5, metric = "cosine", and marker genes were identified using *getMarkerFeatures* with bias = c("TSSEnrichment", "log10(nFrags)"), testMethod = "wilcoxon".

Integration of scRNA-seq and scATAC-seq Data

Single cell ATAC-seq data were integrated with scRNA-seq data using ArchR. Cells from scATAC-seq are directly aligned with cells from scRNA-seq by comparing the scATAC-seq gene score matrix with the scRNA-seq gene expression matrix. This alignment is performed using the *FindTransferAnchors()* function from Seurat, which allows for the alignment of data across two datasets. This cross-platform linkage is performed serially in an unconstrained and constrained fashion. Pseudo-scRna-seq profiles are then generated for each single ATAC cell using the native ArchR *addGeneIntegrationMatrix()* function with default parameters, and scATAC-seq clusters were then labeled with scRNA-seq information. Pseudo-bulk replicates were then generated using

ArchR's *addGroupCoverages()*, permitting peak calling with ArchR's native TileMatrix algorithm. Motif enrichment calculations were performed using *addMotifAnnotations* and p*eakAnnoEnrichment* with cutOff = "FDR <= 0.1 & Log2FC >= 0.5".

GREAT Analysis

Genomic Regions Enrichment of Annotations Tool (GREAT) analysis was performed programmatically in R (rGREAT v1.18.0) to assess for enrichment of cis-regulatory regions (17). This was performed separately for the initial six ATAC-defined clusters and the subsequent four clusters defined based on integrated scRNA-seq analysis. In each case, this was performed iteratively for each cluster using pairwise comparisons between peak sets for that cluster against a background containing the union of all peak sets from the dataset (including those for that cluster).

Deconvolution of Bulk RNA-seq Profiles

CIBERSORTx(18) was used to deconvolve cell type abundances from bulk RNA-seq profiles of fibroblasts isolated from untreated wounds at POD7 (n = 4) and POD14 (n = 3) and FAK-inhibitor-treated wounds at POD14 (n = 4). Default parameters from the web toolkit (http://cibersortx.stanford.edu/) were used to generate a custom signature matrix using a single cell reference matrix file consisting of raw mRNA counts for each cell in the scRNA-seq dataset. Each scRNA cell was re-labeled using Seurat's anchor-transfer mapping algorithm to the integrated ArchR scRNA-ATAC reference profiles: "ArchR-Cluster 1", "ArchR-Cluster 2", "ArchR-Cluster 3", and "ArchR-Cluster 4" subpopulations as defined in **Fig. 3b**. Imputation of cell fractions was then performed using this matrix in conjunction with a mixture file of bulk RNA-seq samples, using S-mode batch correction without quantile normalization.

Spatial Transcriptomics

Wound specimens were rapidly harvested and flash frozen in OCT. Using the Visium Tissue Optimization Slide & Reagent kit, permeabilization time was optimized at a thickness of 10um per section and 37 minutes for mouse cutaneous scar tissue. Wound tissues were cryosectioned at -20 degrees onto gene expression slides. The Gene Expression Slide & Reagent kit was followed per protocol, and used to produce sequencing libraries. The libraries were then sequenced using NextSeq (Illumina), and Bcl files were demultiplexed.

Raw FASTQ files and histology images were processed by sample with the Space Ranger software, which uses STAR v.2.5.1b (Dobin et al., 2013) for genome alignment, against the Cell Ranger mm10 reference genome, available at: http://cf.10xgenomics.com/supp/cell-exp/. Raw spaceranger output files for each sample were read into a Seurat class object in R using Seurat's Load10X in a manner that kept them paired with the low resolution histology images for visualization purposes. This included information such as the number of estimated cell counts, the sum of UMIs per spot, number of expressed genes per spot, and graph-based clustering results. We did not drop any spots given the spatial pattern they presented. Data were normalized using the SCTransform with default parameters. Principal component analysis was performed on the normalized dataset, and the top 30 components were used for neighbor finding, Louvain-based cluster analysis, and UMAP dimension reduction and embedding.

For the delineation of cell type contributions to each Visium "spot", high resolution histology images (H&E) were loaded and each spot was counted for the number of a given cell type in that spot. This was performed by co-authors who were not involved in the Visium data analysis. A maximum of 9 cells of a given type were used as the limit for a given spot. Spots consisting entirely of one cell type, based on these annotations, were used to construct sets of "pure" keratinocyte, endothelial cell, macrophage, and "neutrophil" (including all non-macrophage immune cells) transcriptional profiles. We then sampled from these in silico population in a Monte Carlo fashion using 10,000 iterations to "subtract out" the contribution of non-fibroblast cells from each fibroblast-containing spot in a linear fashion. Negative gene expression values were zeroed

out at a per-spot level following all subtractions, representing an asymmetrical bias for spots based on the number of non-fibroblast cells, which we found to counterbalance the assumption of linear added effects. These values were propagated forward for anchor-based integration to spatially overlaid partial memberships for each of our four clusters. The resulting partial membership contributions for each spot were then averaged to obtain consensus cluster representations

Imputed Spatial Epigenomics

In order to impute spatial epigenomic properties from our Visium datasets, we first re-partitioned our scRNA-ATAC construct with higher cluster resolution in order to better capture differences in the time and space phenotypes represented by the underling scRNA and scATAC data. This parameterization was optimized using our POD 14 spatial transcriptomic data, which had the clearest delineation of dermal margins and highest representation of fibroblasts. We first defined an “outer” ←→ “inner” vector along the scar dermis and then computed the spatial auto-correlation for each gene along this vector using six neighbors for each Visium spot (**Fig. S17A**) (19). We considered the top 100 most highly auto-correlated genes to have spatial significance in our original dataset (**Fig. S17B**). We then iteratively re-partitioned our multimodal scRNA-ATAC fibroblast data using ArchR’s native Louvain clustering algorithm while varying the resolution parameter from 0.5 to 4.0 at increments of 0.1. Each of the resulting cluster configurations was used to generate partial membership profiles for each Visium spot using the anchor-based transfer method described above, after which the Gene Integration Matrix values associated with each ArchR cluster were projected onto each Visium spot in accordance with the each cluster’s partial spot membership. Preservation of auto-correlation for the 100 genes determined above was used as an optimization metric, and we selected a resolution parameter of 2.2, which produced twenty clusters, each representing between 27 and 552 cell equivalents. Gene integration matrix distributions, informed by both modalities, were then extracted for each partition

and subjected to SCT transformation. “Spike-in” scRNA-seq data for keratinocytes, endothelial cells, macrophages, and neutrophils were generated as described above and augmented to the initial 20 RNA-ATAC-defined clusters following a similar SCT transformation. The resulting putative single cell gene expression reference matrix (comprising 24 cell-types) was then used to assign initial partial set memberships for each spatial transcriptomic datapoint using an anchor transfer-based approach. Given that the Visium technology captures multiple cells within each 55 um spot, and discretizes all of their location to the spatial center of that spot, we applied a single-step spatial smoothing function to incorporate fractional membership contributions from each of the six neighboring spots. This is achieved by updating each partial membership value m_i for each $spot_j$ in the Visium slide:

$$m_i(spot_j) = \frac{1}{2} m_i(spot_j) + \frac{1}{12} \sum_{\substack{k\in \\ neighbor(j)}} m_i(spot_k)$$

Partial memberships to non-fibroblast clusters were subsequently removed from each spot, and the remaining partial membership values for each fibroblast cluster were normalized to enforce a membership sum of 1 for each spot:

$$m_i(spot_j) = m_i(spot_j) \frac{\sum_{\forall k} m_k(spot_j)}{\sum_{k\in fibroblast} m_k(spot_j)}$$

The resulting partial set memberships for each spatial datapoint were then treated as a topological vector space, onto which epigenomic peak, motif, and binding activity from the twenty scRNA-ATAC partitions can be projected.

To examine the spatial dependence of individual genes along the out-to-inner wound axis, spatial lag vectors were constructed for POD 14 Visium samples using a 3-spot diameter trajectory as shown in (**Fig. S17A**) (19), and Pearson correlations calculated for imputed gene scores along either radial direction. The top 200 genes from each group were used to perform enrichment analysis against the Gene Ontology database. Functional networks of the most highly enriched gene sets were then generated using the clusterProfiler package in R (20).

Statistical Analysis

Non-omics statistical analyses were performed using the software GraphPad Prism v.6 (unless otherwise noted). Results are expressed as absolute numbers, percentages, fractions, or mean +/- standard deviation (unless otherwise noted). Unpaired *t*-test assuming two-tailed distribution or one-way analysis of variance (ANOVA) and post hoc Tukey correction were used to compare groups where relevant. $P < 0.05$ was considered statistically significant. All -omics statistical analyses were performed in R (version 3.6.0) as described above.

SUPPLEMENTAL FIGURES

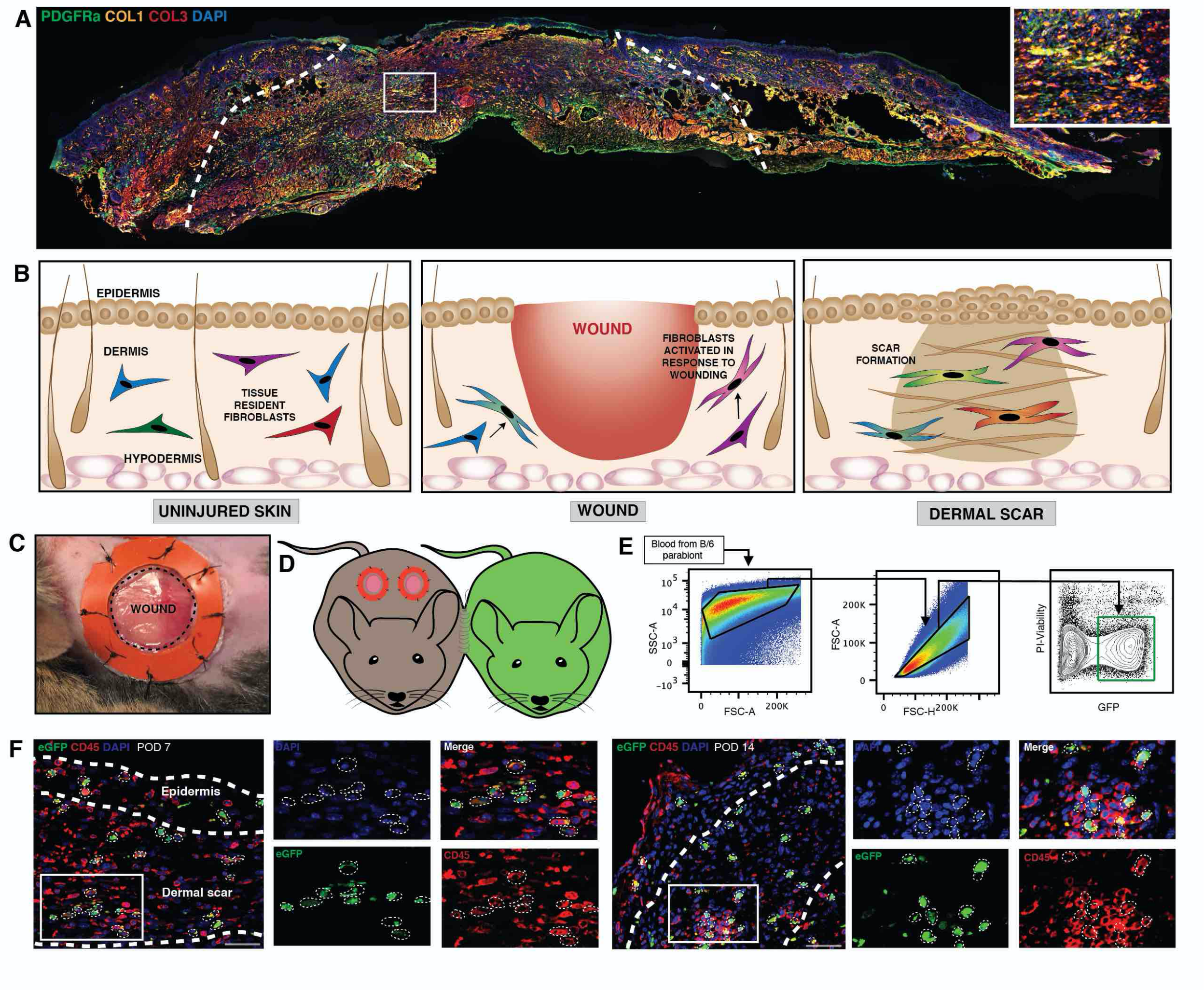

Supplemental Figure 1.

A, Representative stitched image of a complete mouse wound specimen on cross section immuno-stained for expression of PDGFRA, COL1, and COL3, with DAPI for nuclei. White dotted lines indicate wound scar edge. Solid box indicates zoom of region indicated on figure panel. *n* = 3 per condition.

B, Schematic illustrating how tissue-resident fibroblasts (**left panel**) are activated in response to wound injury (**middle panel**) and contribute to scar fibrosis (**right panel**).

C, Representative photograph showing a fresh wound prepared using the stented dorsal wound healing model employed in this study, which limits contraction of the panniculus carnosus and

thereby mimics human wound healing kinetics (1). Black dotted line circumscribes the wound edge.

D, Schematic of the mouse parabiosis model used, with dorsal wounds created on the wild-type (Black/6) mouse parabiosed to an eGFP mouse after shared blood supply has been established between the parabionts.

E, Representative FACS-plots showing analysis of blood taken from a wild-type (Black6) parabiont 2 weeks following parabiosis to an eGFP mice. Establishment of a shared blood supply is indicated by the presence of circulating GFP+ cells (green box).

F, Representative histologic sections from wounds made on the wild-type parabiont and harvested at post-operative day (POD) 7 (**left panels**) and POD 14 (**right panels**), demonstrating systemically-derived (GFP+) cells within the scar area; the vast majority of these cells co-stain for CD45. DAPI (blue) identifies cell nuclei. Thick white dotted lines indicate dermal scar area, and thin white dotted lines highlight individual GFP+ DAPI+ cells. Right panels correspond to the sub-regions outlined by white solid boxes. *n* = 3 per condition per timepoint. Scale bar = 25μm.

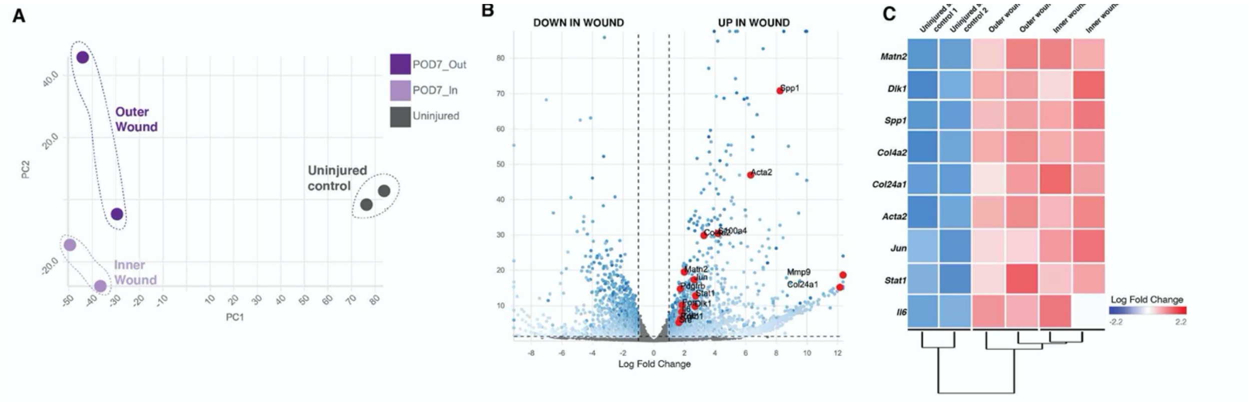

Supplemental Figure 2.

A, PCA plot of bulk RNA-seq of wound specimens harvested at POD 7 showing clustering of inner wound region fibroblast gene expression as distinct from the corresponding outer wound region fibroblasts and uninjured control skin fibroblasts.

B, Volcano plot highlighting genes of interest (pro-fibrotic and mechanotransduction-related) upregulated in wound conditions compared with fibroblasts isolated from uninjured control skin from bulk RNA-seq displayed above**.**

C, Selected heatmap displaying hierarchical clustering for inner wound region fibroblasts compared with outer and uninjured control skin and highlighting relative expression of specific fibrosis- and mechanotransduction-related genes among the aforementioned fibroblast samples. Scale at right represents log fold-change.

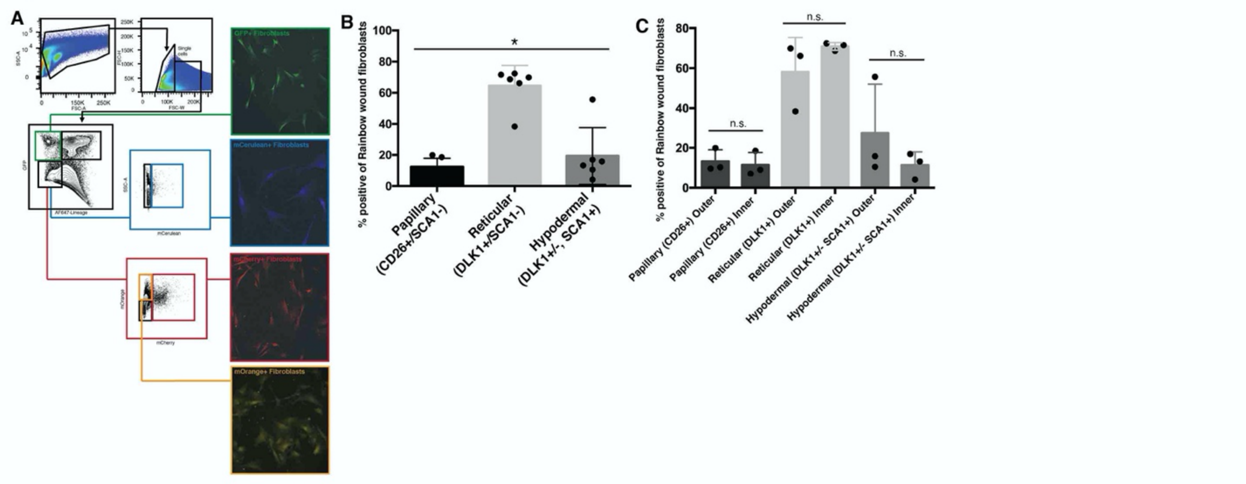

Supplemental Figure 3.

A, Representative FACS-plots showing isolation of Rainbow wound fibroblasts. Images at right show plated, freshly-isolated Rainbow fibroblasts for morphological and fluorophore-expression validation. Rainbow colors as labelled in figure.

B, Flow cytometry analysis of FACS-isolated Rainbow wound fibroblasts for expression of cell surface markers associated with skin fibroblast subpopulations. *n* = 6.

C, Flow cytometry analysis for expression of cell surface markers associated with skin fibroblast subpopulations of FACS-isolated Rainbow wound fibroblasts separated into inner and outer wound regions. *n* = 3.

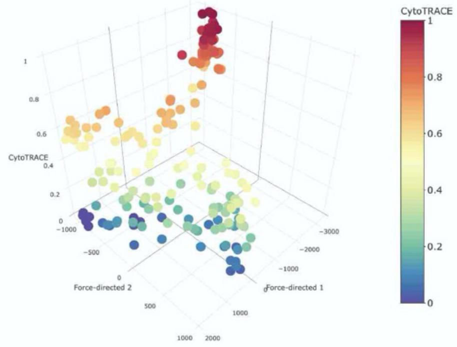

Supplemental Figure 4.

3D embedding of CytoTRACE data shown in **Fig. 2D** further delineates cells based on predicted differentiation capacity.

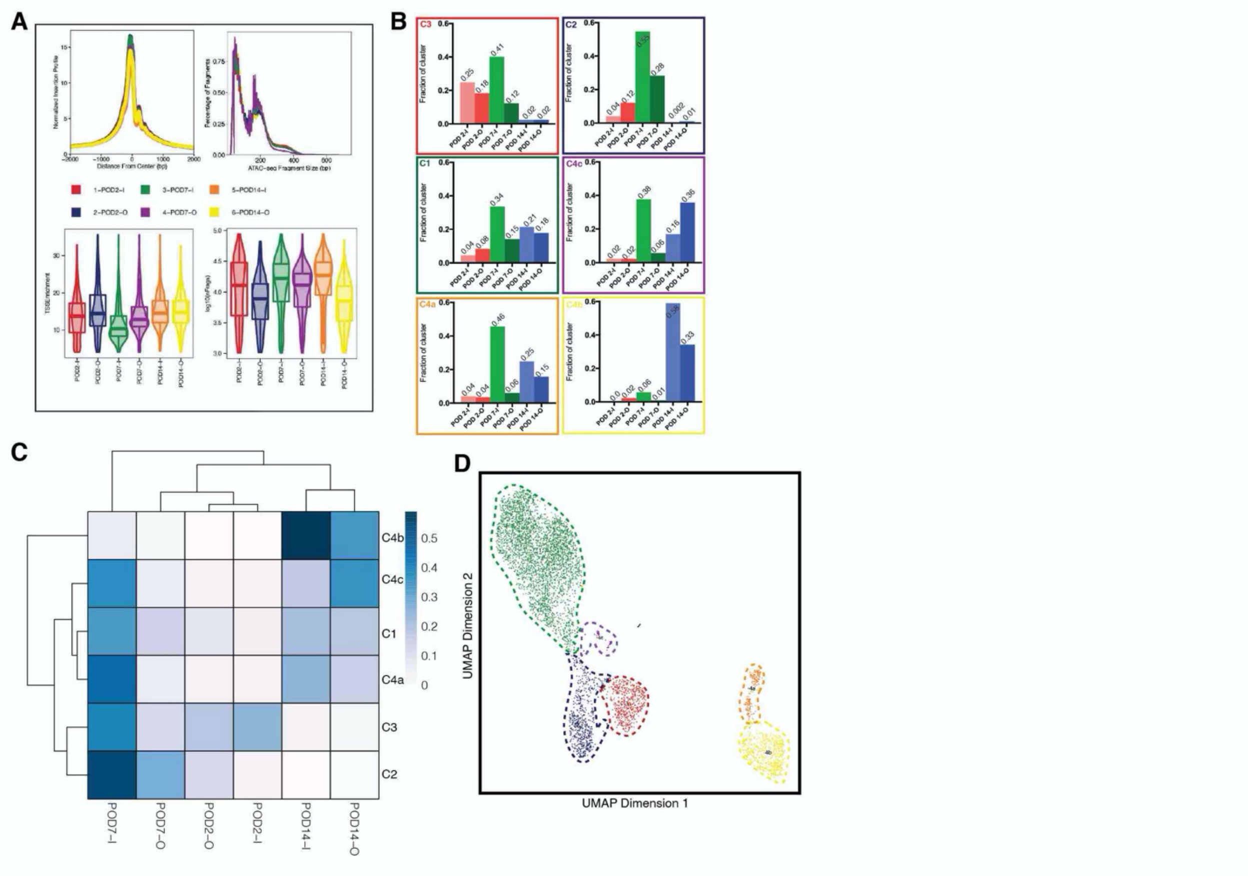

Supplemental Figure 5.

A, Overlay of transcription start site (TSS) enrichment and fragment enrichment for scATAC-seq samples (**top panels**). Violin plots showing TSS and fragment enrichment for each sample type analyzed by scATAC-seq (**bottom panels**). POD = post-operative day, “O” = Outer wound region, “I” = Inner wound region.

B, Breakdowns of isolation time point and inner versus outer region status are provided for each scATAC-seq cluster. Y-axis displays cell fraction of cluster total for each of the 6 scATAC-seq conditions; individual fraction values are noted above each bar.

C, Heatmap with hierarchical clustering highlighting relationships between scATAC-seq specimens based on phenotype and cluster assignment. POD = post-operative day, I = inner, O = outer.

D, scATAC-seq evaluation of Rainbow mouse wound fibroblasts isolated in parallel with our scRNA-seq experiments (see Methods). UMAP embeddings were generated using ArchR with default Louvain parameters(21), and six unique fibroblast clusters were identified.

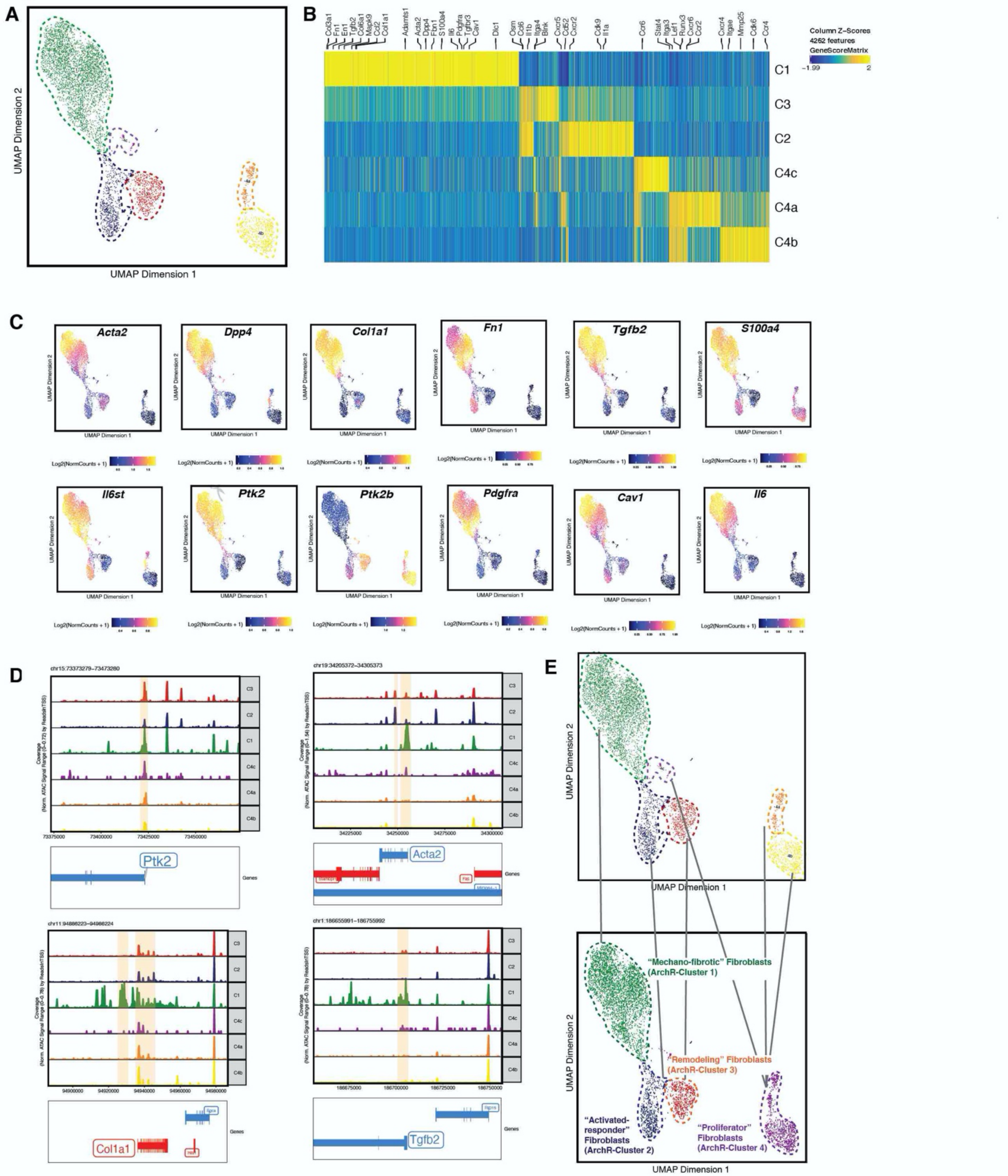

Supplemental Figure 6.

A, UMAP embedding of scATAC-seq fibroblasts showing six epigenomically-defined clusters, provided for reference.

B, Heatmap of scATAC-seq cluster peaks mapped to associated gene markers. Elements relevant to fibroblast activation, fibrosis, and mechanotransduction are specifically annotated along the top of panel.

C, UMAP feature plots highlighting distributions for genes of interest related to fibrosis and mechanotransduction.

D, Genome tracking plots showing scATAC-seq peaks for pseudo-bulk replicates generated for each cluster. Associations between the peaks with fibrosis and mechanotransduction-related genes (Peak2GeneLinks) are included at the bottom of each plot. Pale orange shading highlights differentially expressed peaks across the scATAC Clusters. All highlighted peaks demonstrated statistically significant differential expression in at least one pairwise comparison (FDR < 0.1 and FC >= 2).

E, Mapping of scATAC-only defined clusters to integrated scRNA-ATAC clusters.

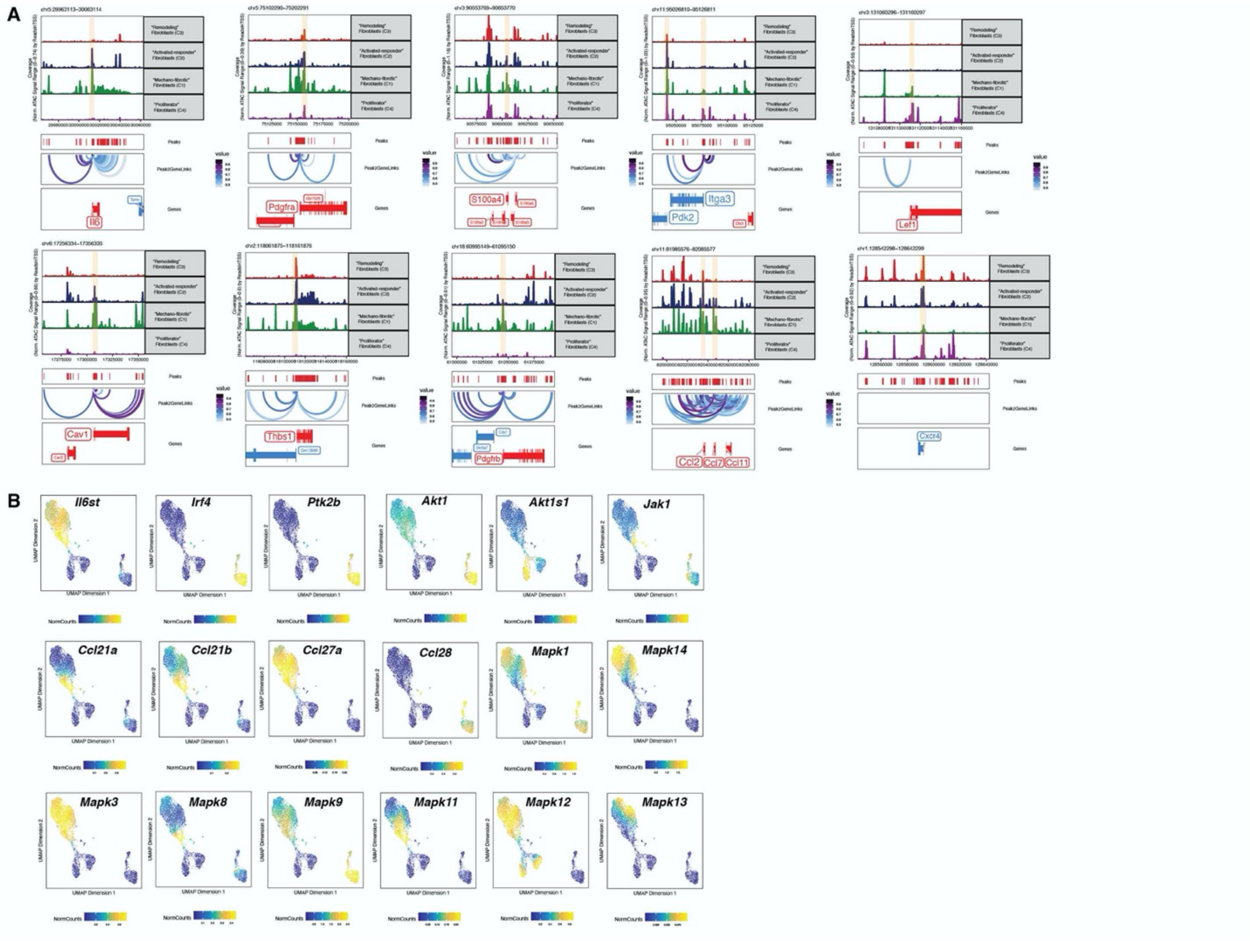

Supplemental Figure 7.

A, Additional tracking plots showing integrated ArchR peaks per cluster at top of each plot, and association between the peaks with fibrosis and mechanotransduction-related genes (Peak2GeneLinks) at the bottom of each plot. Pale orange shading highlights differentially expressed peaks across the ArchR-Clusters. All highlighted peaks demonstrated statistically significant differential expression in at least one pairwise comparison with FDR < 0.1 and FC >= 2.

B, Additional feature plots showing integrated ArchR chromatin accessibility and gene expression data for the ArchR-Clusters from A in terms of fibrosis- and mechanotransduction-related genes of interest.

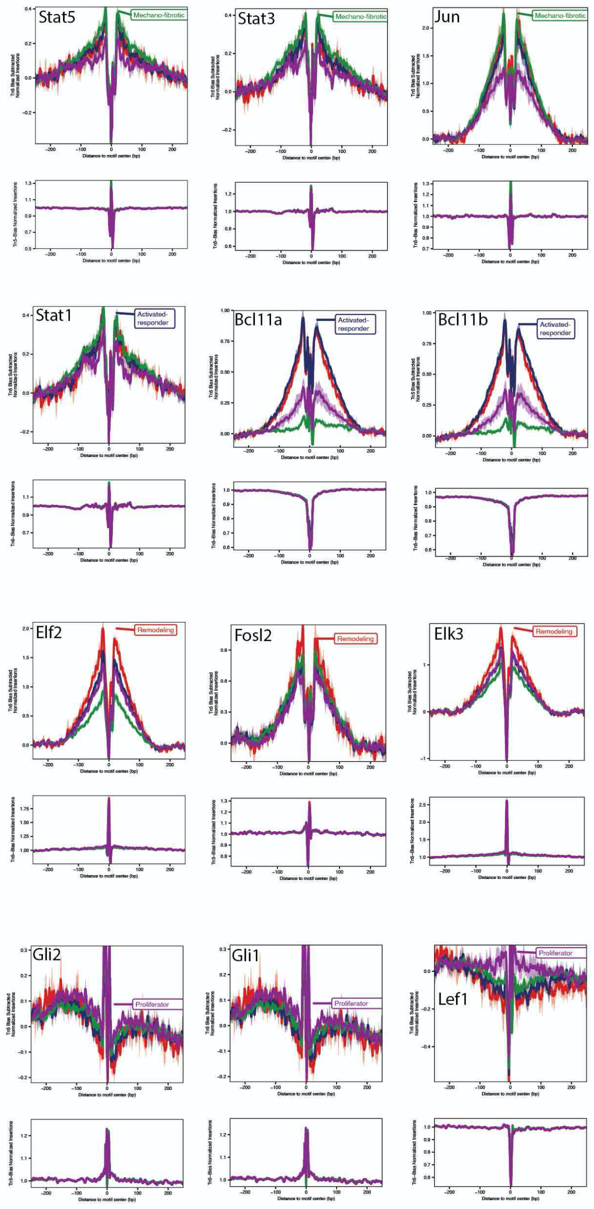

Supplemental Figure 8.

Notable transcription factor (TF) footprinting based on accessibility at a given motif across all identified ATAC peaks, from the integrated ArchR dataset based on pseudo-bulk peak calls. Top row highlights TF footprinting plots relevant for ArchR-Cluster 1, second row for ArchR-Cluster 2, third row for ArchR-Cluster 3, and fourth row for ArchR-Cluster 4.

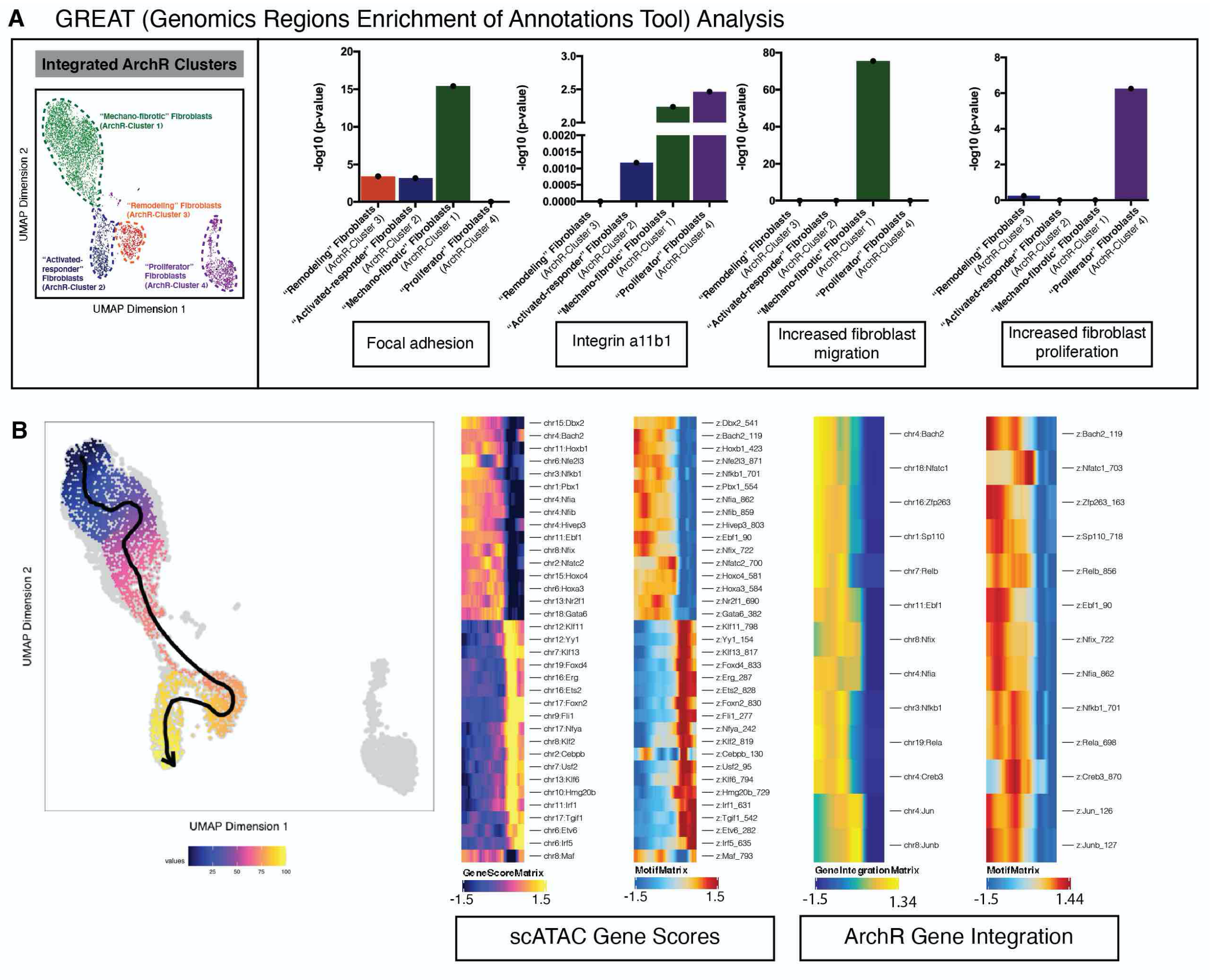

Supplemental Figure 9.

A, GREAT analysis of ArchR Cluster data showing enrichment for specific gene sets of interest.

B, ArchR-generated pseudotime plot using standard UMAP embedding (left panel), and heatmaps showing Gene Score values imputed from chromatin peak accessibility (middle panels) and Gene Integration values imputed from integration of scRNA-seq data (right panels) relative to pseudotime.

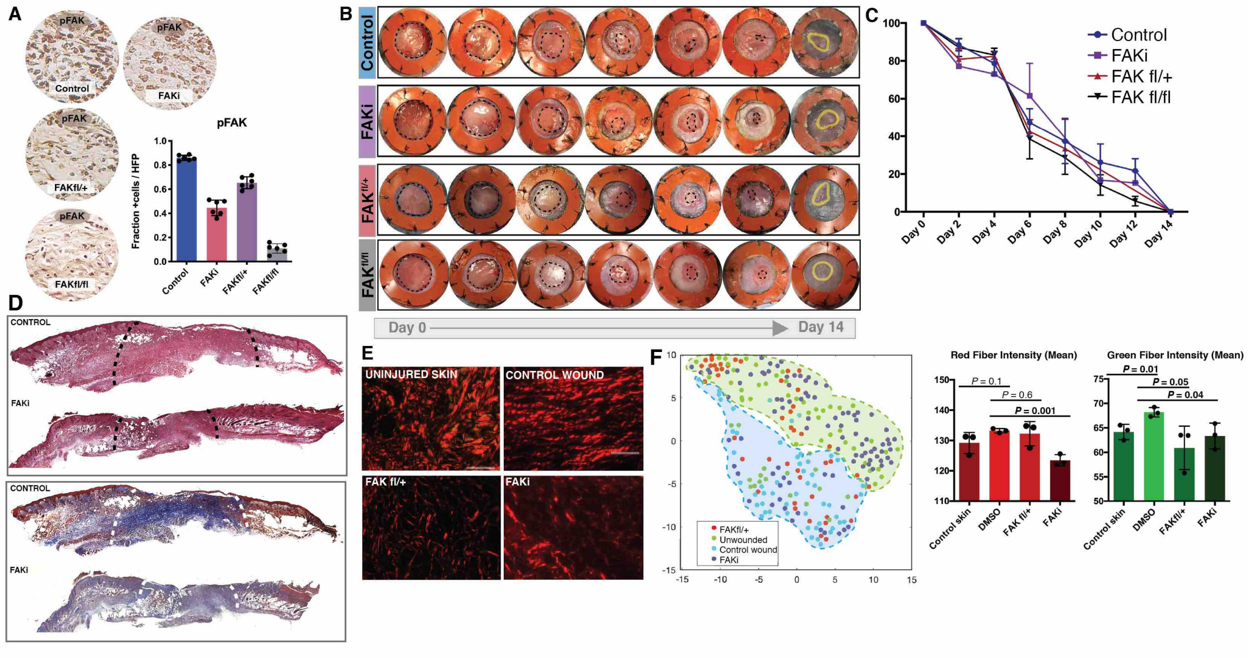

Supplemental Figure 10.

A, Representative DAB staining for expression of pFAK in vehicle control-treated wounds (**left top panel**) compared with FAK$^{fl/+}$ (**left middle panel**) or FAKi-treated specimens (**left bottom panel**). *n* = 3 per condition. Quantitation showing fraction of pFAK+ cells per HPF per condition (**right panel**)

B, Representative photographs of healing wounds taken every other day over 14 days for the following conditions: vehicle control (**top sub-panel, blue**), FAKi (**second sub-panel, purple**), FAK$^{fl/+}$ (**third sub-panel, red**), and FAK$^{fl/fl}$ (**bottom sub-panel, grey**). Dotted black lines indicate open wound area. Solid yellow line highlights the scar area within healed wounds at POD 14.

C, Quantitation of wound healing data from left panels shows no significant difference in rate of wound re-epithelialization among groups.

D, H&E (**top panels**) and trichrome (**bottom panels**) of stitched images of complete wound specimens for vehicle control (top panels) compared with FAKi-treated wounds at POD 14. White dotted lines indicated edge of wound scar area. *n* = 5.

E, Representative picrosirius red staining of wound specimens: uninjured skin (**top left**), vehicle-control wound (**top right**), FAKi-treated wound (**bottom left**), and $FAK^{fl/+}$ wound (**bottom right**). *n* = 3 per condition. Scale bars = 50μm.

F, Automated connective tissue analysis of polarization microscopy images of Picrosirius Red-stained histology specimens shown in (**E**). The connective tissue ultrastructure in FAKi-treated or from $FAK^{fl/+}$ specimens is more similar to unwounded skin than vehicle-control (DMSO) wounds on t-SNE analysis. Sample types highlighted with colored dotted lines (**left panel**). Quantitative analysis of mean red (**middle panel**) and green (**right panel**) fiber intensity for Control (injured) skin, and DMSO (vehicle control), $FAK^{fl/+}$, and FAKi-treated wounds. Statistically significant differences are seen with FAKi-treatment in terms of both fiber types.

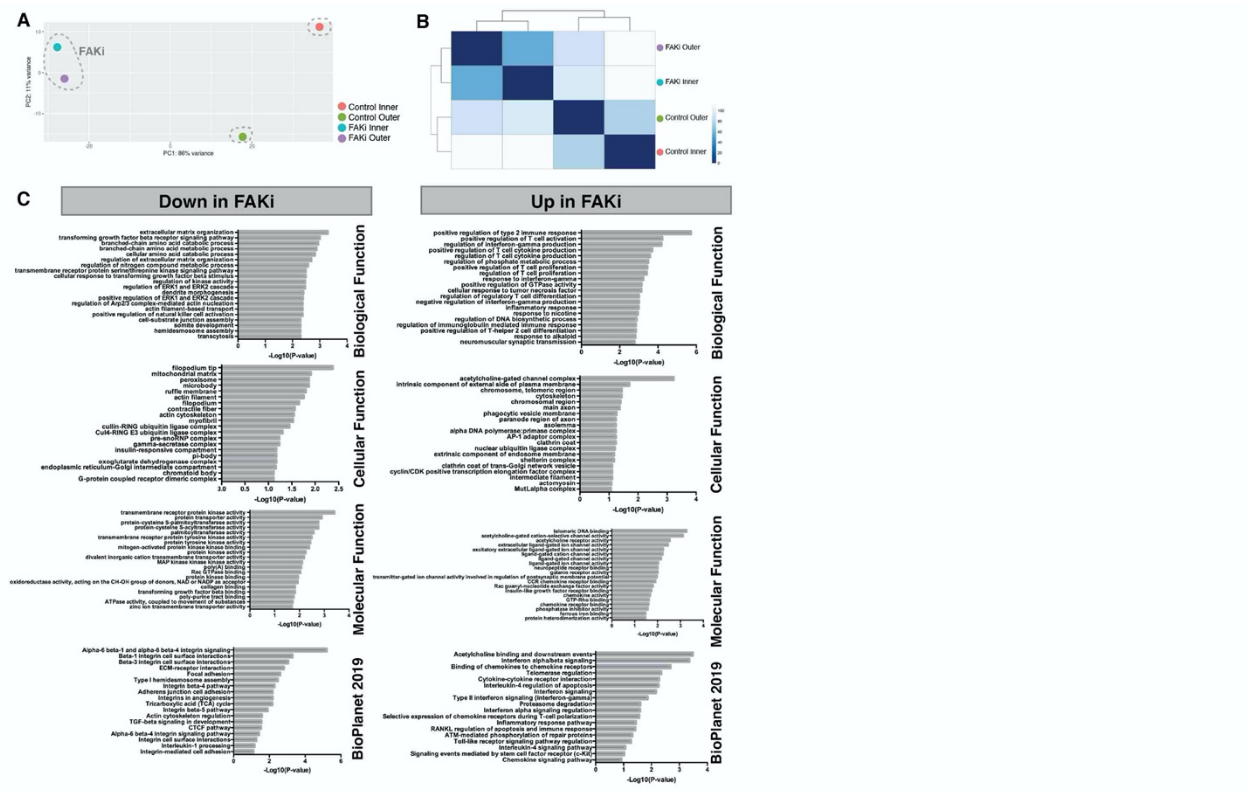

Supplemental Figure 11.

A, PCA-analysis of bulk RNA-seq data for mouse fibroblasts isolated from wounds treated with FAKi versus control and separated in terms of inner and outer wound regions at POD 14.

B, Sample-level heatmap with hierarchical clustering of bulk RNA-seq data from inner and outer wound regions with or without FAKi treatment.

C, EnrichR analysis for bulk RNA-seq data comparing FAKi-treated wound fibroblasts with vehicle-control wounds. Left panels show gene pathways down with FAKi, right panel shows gene pathways up with FAKi. Top three panels show the 20 most significant GO terms for each cell condition (Biological function, Cellular function, Molecular function). Bottom panels show the top 20 pathways from the BioPlanet 2019 database. All enrichment analyses were conducted using the top 100 most significant genes based on adjusted p-value.

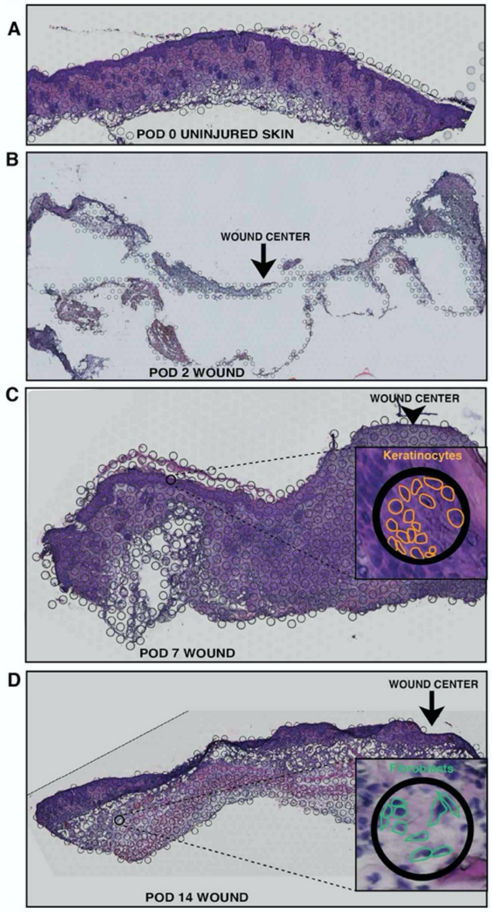

Supplemental Figure 12.

A-D, Manual annotation of cell types within Visium spots based on H&E histology for (**A**) POD 0 (unwounded), (**B**) POD 2, (**c**) POD 7, and (**d**) POD14. Panels to the right of (**C**) show zoomed views for an arbitrary wound region, with the second zoomed panel representing a single Visium spot. Fibroblasts are circled with green and keratinocytes circled with orange.

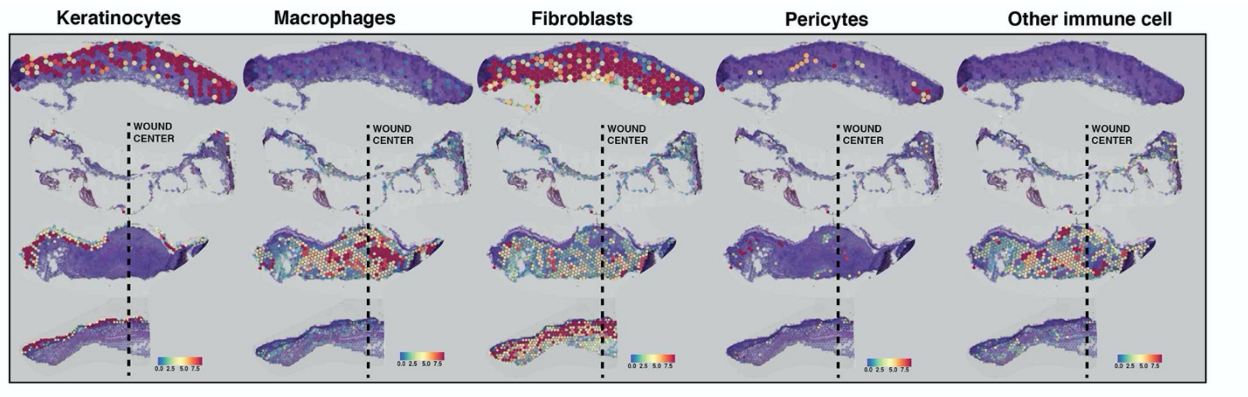

Supplemental Figure 13.

Projection of manual cell type annotations onto Visium slides across all four time points. In this context we reference “other immune” cells in order to reinforce that these assessments are simply estimations and as such are subject to considerable variability.

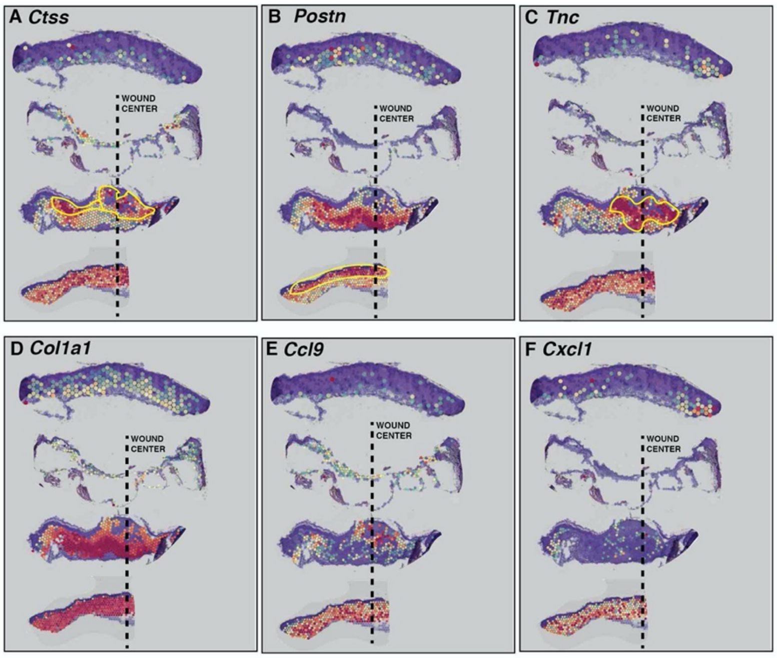

Supplemental Figure 14.

A-F, Visium plots showing POD 0, 2, 7, and 14 (top to bottom) wound sections. Expression of *Ctss*, which is a known mechanoresponsive gene(22), closely aligns with *Spp1*+ *Mmp9*+ Cluster 4 fibroblasts (see **Fig. 4**) along the closing edge at POD7 (**A**). *Postn* expression appears along the dermis later over the wound healing course at POD7 and then localizes to the suprabasal region at POD 14 (**B**), whereas *Tnc* expression appears more in the inner wound at POD7 and then is expressed diffusely across wound fibroblasts at POD14 (**C**). *Col1a1* expression is very prominent across the scar over the course of wound healing (**D**). Expression of *Ccl9* and *Cxcl1* is prominent in the wound dermis particularly at POD14 highlighting how fibroblasts regulate immune cells within the wound microenvironment, similar to *Cxcl5* (see **Fig. 4**) (**E-F**).

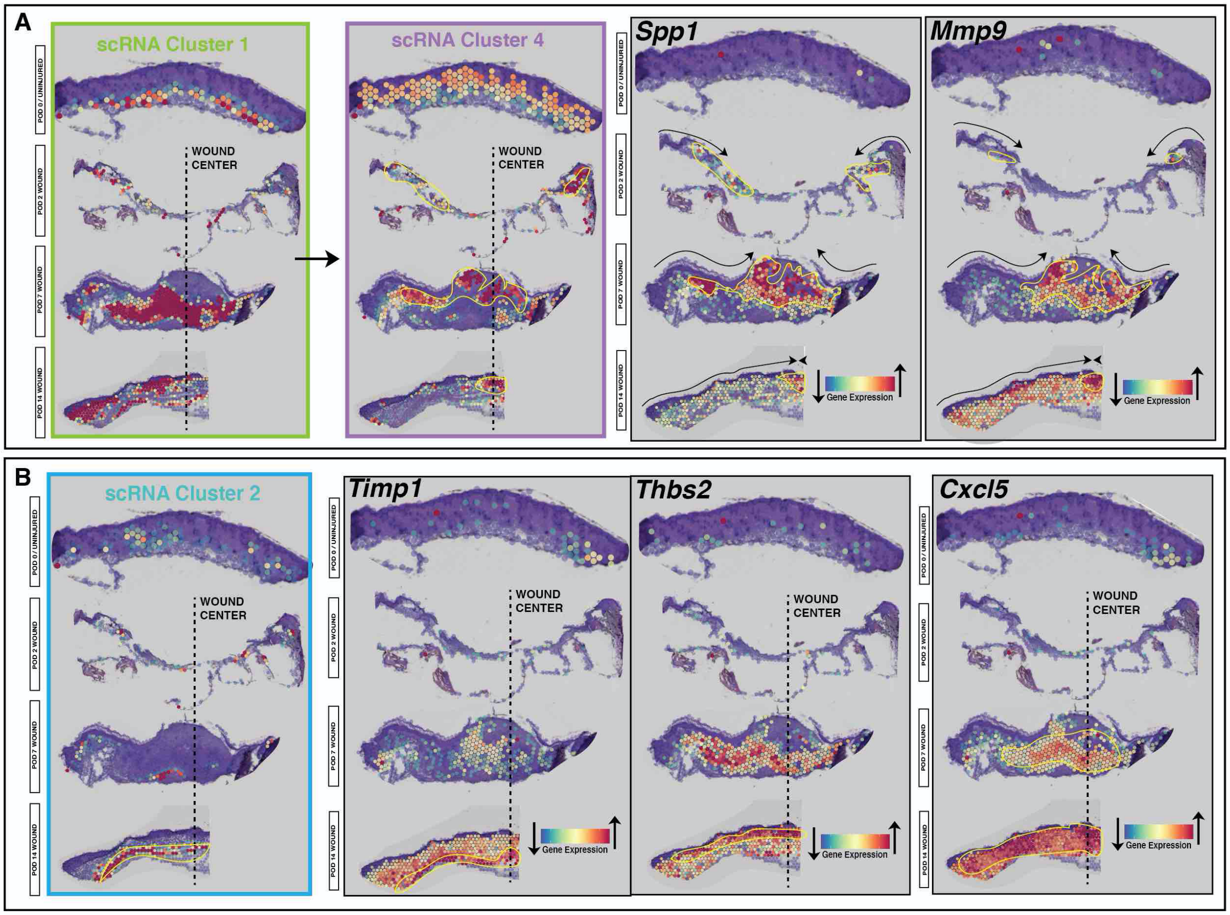

Supplemental Figure 15.

A, Visium plots showing POD 0, 2, 7, and 14 (top to bottom) wound sections. As wound fibroblasts proliferate from the outer region of the wound inwards, a portion of Cluster 1 cells appear to differentiate into Cluster 4 cells. Cluster 4 cells prominently express *Spp1* and *Mmp9*, and track along the same trajectory of the healing edge of the epidermis. Wound centers indicated by dotted line as labelled in panels. Left 2 panels show partial membership clusters from a. Right two panels show SCT gene expression.

B, Visium plots showing POD 0, 2, 7, and 14 (top to bottom) wound sections. Cluster 2 cells appear to populate the basal dermis primarily. Gene expression of *Timp1* highlights this subpopulation, whereas *Thbs1* expression highlights suprabasal wound fibroblasts,

which strongly express chemokines such as *Cxcl5* regulating immune cells within the wound microenvironment. Wound centers indicated by dotted line as labelled in panels. Left panel show partial membership clusters. Right three panels show gene expression data.

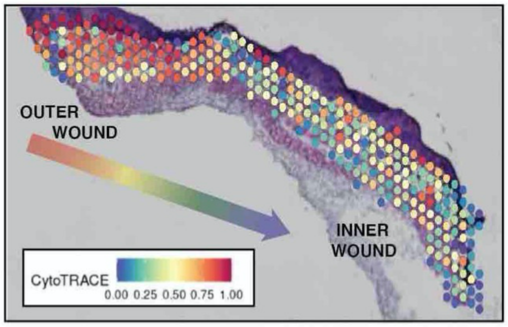

Supplemental Figure 16.

CytoTRACE applied to spatial transcriptomics recapitulates cellular differentiation from the outer to inner regions of the wound.

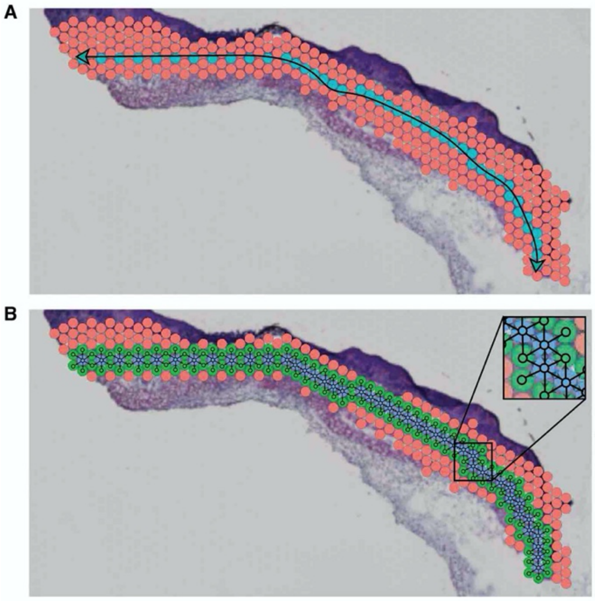

Supplemental Figure 17.

A, “Outer” ←→ “inner” vector along central dermis.

B, Spatial lag vector, encompassing six hexagonal "neighbors” for each Visium spot.

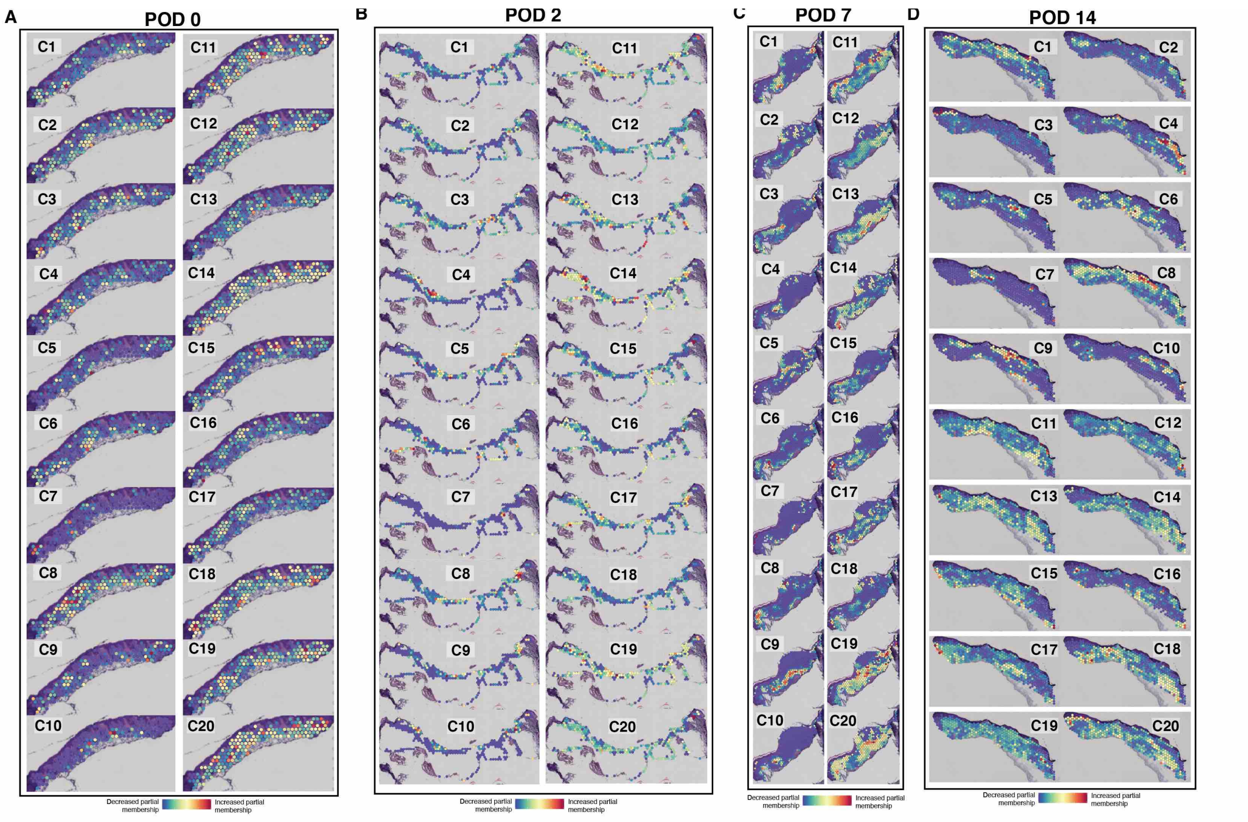

Supplemental Figure 18.

A-D, Overlay of scRNA-ATAC partial probability memberships for each of the twenty partitions onto Visium histology sections at (**A**) POD 0 (unwounded skin), (**B**) POD 2, (**C**) POD 7, and (**D**) POD 14.

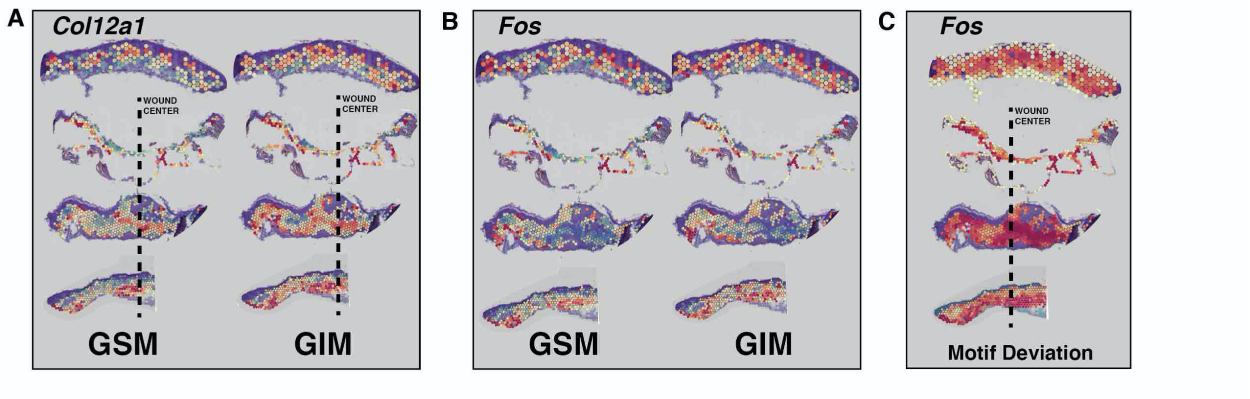

Supplemental Figure 19.

A-C, Visium plots showing POD 0, 2, 7, and 14 (top to bottom) wound sections, imputed spatial epigenomics. **(A)** For housekeeping genes such as *Col12a1* (top panel), gene imputed matrix (GIM) correlates with gene score matrix (GSM) epigenomic data and is fairly stable over space and time. **(B)** However, for the profibrotic gene *Fos*, which is very active within wound fibroblasts, GSM data shows opening at the *Fos* motif along the outer wound at POD2, which yields strong gene expression across wound fibroblasts at POD 7. **(C)** Visium motif deviation plots showing POD 0, 2, 7, and 14 (top to bottom) for *Fos* shows significant openness proximal to the *Fos* motif across the wound area at POD7, likely preceeding gene expression in this area which is present at POD 14.

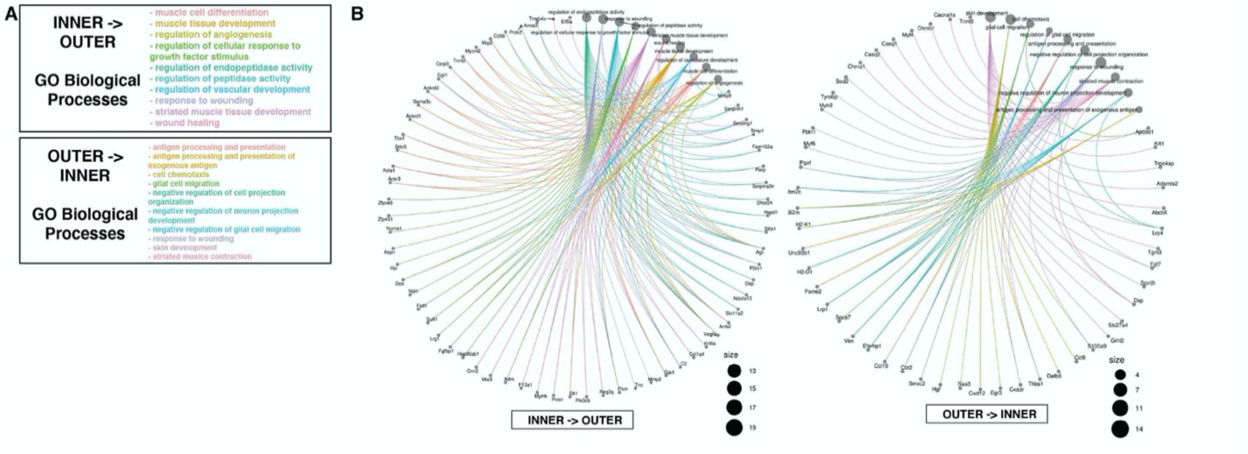

Supplemental Figure 20.

A-B, Using the spatial lag vector described in **Supplemental Fig. 17**, Pearson correlations were calculated for epigenomically-imputed chromatin accessibility on a per-gene basis along either radial direction. The most highly correlated genes from each direction (**B**) were then used to generate functional enrichment plots using the Gene Ontology (GO) database (**A-B**).

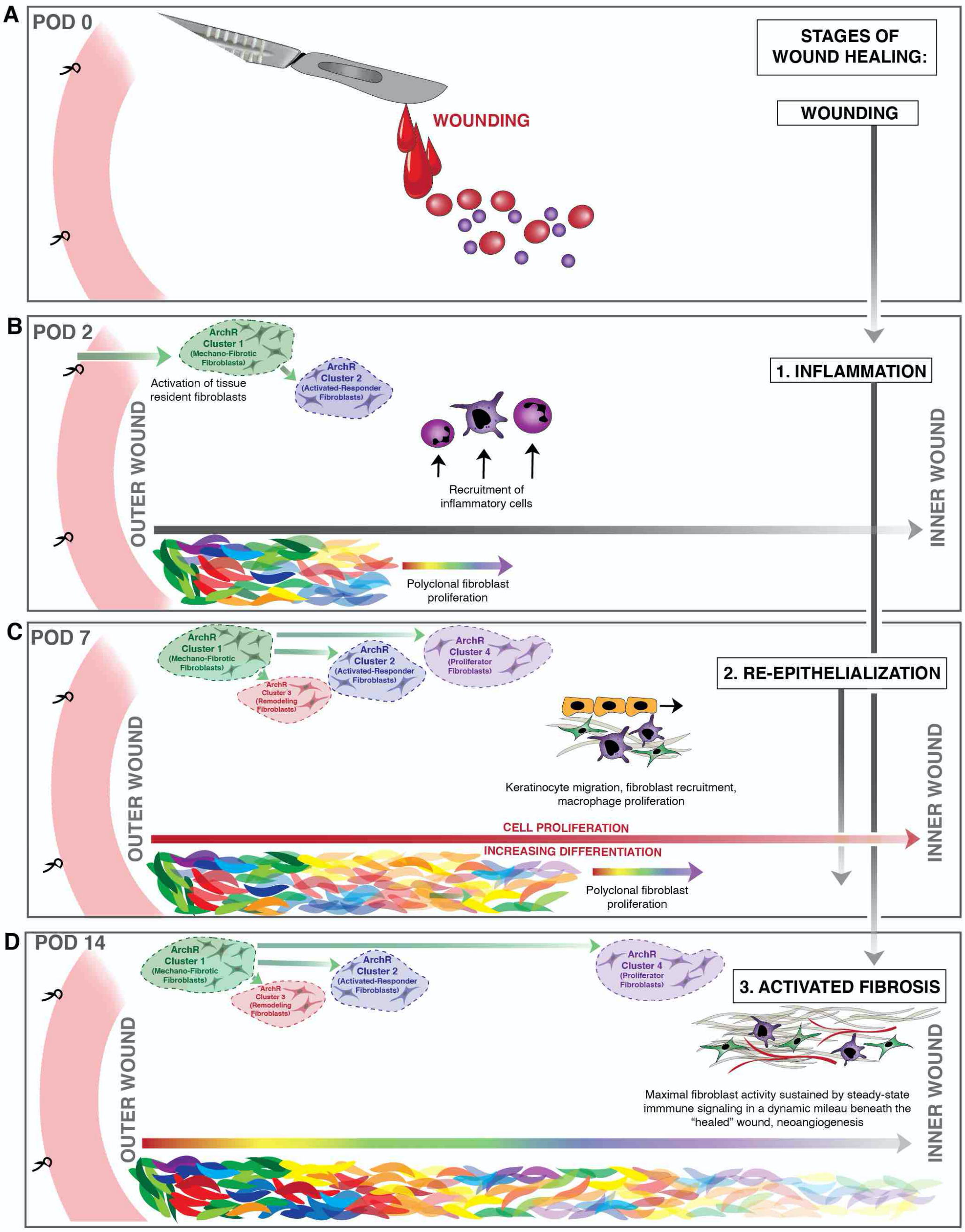

Supplemental Figure 21.

A-D, Vogelgram for wound healing.

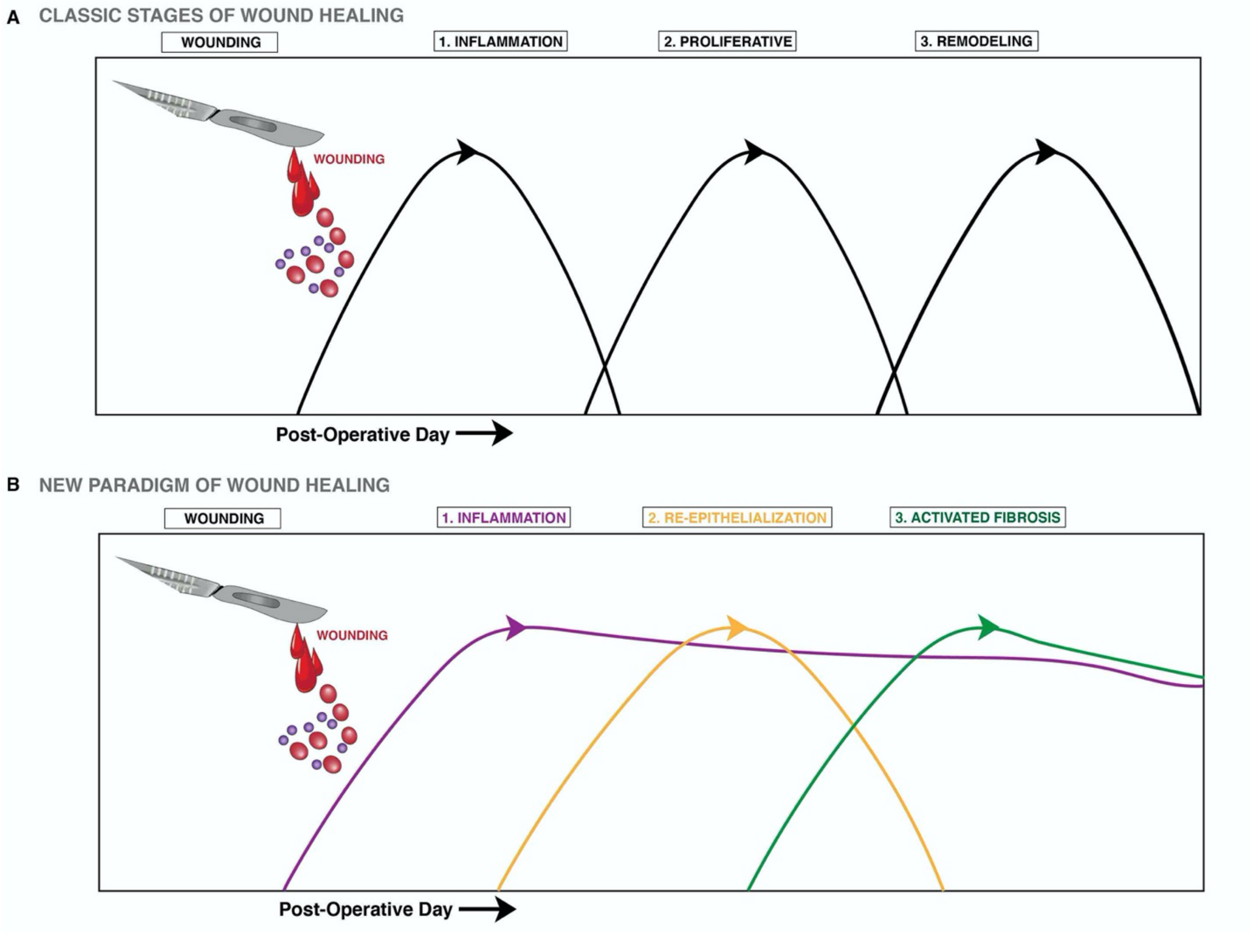

Supplemental Figure 22.

A-B, The findings presented here challenge the classical stages of wound healing (**A**), typically described as three largely discrete phases: inflammation (POD 2), proliferation (POD 7), and remodeling (POD 14). We propose a new framework for viewing the stages of wound healing: 1) Early inflammation, 2) Re-epithelialization, and 3) Activated fibrosis – where maximal fibroblast activation is achieved and sustained by steady-state inflammatory signaling beneath the "healed" wound (**B**).

	C1	C2	C3	C4	C5	C6	C7	C8	C9	C10	C11	C12	C13	C14	C15	C16	C17	C18	C19	C20
**ArchR Cluster 1**	0	0	0	0	0	162	27	4	0	0	381	450	407	503	253	201	340	552	0	0
**ArchR Cluster 2**	0	0	0	0	0	0	0	204	27	0	0	0	0	0	0	0	0	0	418	0
**ArchR Cluster 3**	0	0	0	0	0	0	0	4	0	0	0	0	0	0	0	0	0	0	0	533
**ArchR Cluster 4**	181	247	209	107	90	0	0	0	25	28	0	0	0	0	0	0	0	0	0	0

Supplemental Table 1.

SUPPLEMENTAL REFERENCES

1. R. D. Galiano, J. t. Michaels, M. Dobryansky, J. P. Levine, G. C. Gurtner, Quantitative and reproducible murine model of excisional wound healing. *Wound Repair Regen* **12**, 485-492 (2004).

2. P. Kamran *et al.*, Parabiosis in mice: a detailed protocol. *J Vis Exp* 10.3791/50556 (2013).

3. R. C. Ransom *et al.*, Genetic dissection of clonal lineage relationships with hydroxytamoxifen liposomes. *Nat Commun* **9**, 2971 (2018).

4. D. S. Foster *et al.*, A Clearing Technique to Enhance Endogenous Fluorophores in Skin and Soft Tissue. *Sci Rep* **9**, 15791 (2019).

5. E. Rognoni *et al.*, Fibroblast state switching orchestrates dermal maturation and wound healing. *Mol Syst Biol* **14**, e8174 (2018).

6. M. S. Hu *et al.*, Gene expression in fetal murine keratinocytes and fibroblasts. *J Surg Res* **190**, 344-357 (2014).

7. G. G. Walmsley *et al.*, Murine Dermal Fibroblast Isolation by FACS. *J Vis Exp* 10.3791/53430 (2016).

8. R. Patro, G. Duggal, M. I. Love, R. A. Irizarry, C. Kingsford, Salmon provides fast and bias-aware quantification of transcript expression. *Nat Methods* **14**, 417-419 (2017).

9. C. Soneson, M. I. Love, M. D. Robinson, Differential analyses for RNA-seq: transcript-level estimates improve gene-level inferences. *F1000Res* **4**, 1521 (2015).

10. S. Picelli *et al.*, Full-length RNA-seq from single cells using Smart-seq2. *Nat Protoc* **9**, 171-181 (2014).

11. S. Mora-Castilla *et al.*, Miniaturization Technologies for Efficient Single-Cell Library Preparation for Next-Generation Sequencing. *J Lab Autom* **21**, 557-567 (2016).

12. T. Stuart *et al.*, Comprehensive Integration of Single-Cell Data. *Cell* **177**, 1888-1902 e1821 (2019).

13. E. Becht *et al.*, Dimensionality reduction for visualizing single-cell data using UMAP. *Nat Biotechnol* 10.1038/nbt.4314 (2018).

14. N. R. Clark *et al.*, The characteristic direction: a geometrical approach to identify differentially expressed genes. *BMC Bioinformatics* **15**, 79 (2014).

15. E. Y. Chen *et al.*, Enrichr: interactive and collaborative HTML5 gene list enrichment analysis tool. *BMC Bioinformatics* **14**, 128 (2013).

16. G. S. Gulati *et al.*, Single-cell transcriptional diversity is a hallmark of developmental potential. *Science* **367**, 405-411 (2020).

17. C. Y. McLean *et al.*, GREAT improves functional interpretation of cis-regulatory regions. *Nat Biotechnol* **28**, 495-501 (2010).

18. A. M. Newman *et al.*, Determining cell type abundance and expression from bulk tissues with digital cytometry. *Nat Biotechnol* **37**, 773-782 (2019).

19. J. Bergenstrahle, L. Larsson, J. Lundeberg, Seamless integration of image and molecular analysis for spatial transcriptomics workflows. *BMC Genomics* **21**, 482 (2020).

20. G. Yu, L. G. Wang, Y. Han, Q. Y. He, clusterProfiler: an R package for comparing biological themes among gene clusters. *OMICS* **16**, 284-287 (2012).

21. J. M. Granja *et al.*, ArchR is a scalable software package for integrative single-cell chromatin accessibility analysis. *Nat Genet* **53**, 403-411 (2021).

22. M. Januszyk *et al.*, The Role of Focal Adhesion Kinase in Keratinocyte Fibrogenic Gene Expression. *Int J Mol Sci* **18** (2017).
